# Chromatin Topological Domains Associate With the Rapid Formation of Tandem Duplicates in Plants

**DOI:** 10.1002/advs.202408861

**Published:** 2024-12-27

**Authors:** Ni Ma, Xiaopeng Li, Dong Ci, Hai Yue Zeng, Congxiao Zhang, Xiaodong Xie, Caihong Zhong, Xing Wang Deng, Dawei Li, Hang He

**Affiliations:** ^1^ School of Advanced Agriculture Sciences and School of Life Sciences, Academy for Advanced Interdisciplinary Studies, State Key Laboratory of Protein and Plant Gene Research Peking University Beijing 100871 China; ^2^ Peking University Institute of Advanced Agricultural Sciences Shandong Laboratory of Advanced Agricultural Sciences in Weifang Shandong 261325 China; ^3^ Key Laboratory of Plant Germplasm Enhancement and Specialty Agriculture, Wuhan Botanical Garden The Chinese Academy of Sciences Wuhan Hubei 430074 China

**Keywords:** chromatin organization, compartmental domain, rabl configuration, tandem duplication, topologically associating domain

## Abstract

In eukaryotes, chromatin is compacted within nuclei under the principle of compartmentalization. On top of that, condensin II establishes eukaryotic chromosome territories, while cohesin organizes the vertebrate genome by extruding chromatin loops and forming topologically associating domains (TADs). Thus far, the formation and roles of these chromatin structures in plants remain poorly understood. This study integrates Hi‐C data from diverse plant species, demonstrating that nuclear DNA content influences large‐scale chromosome conformation and affects the finer details of compartmental patterns. These contrasting compartmental patterns are distinguished by gene‐to‐gene loops and validated through cytological observations. Additionally, a novel chromatin domain type associated with tandem duplicate gene clusters is identified. These domains are independent of H3K27me3‐mediated chromatin compartmentalization and exhibit evolutionary conservation across species. Gene pairs within TAD‐like domains are younger and show higher levels of coexpression. These domains potentially promote the formation of tandem duplicates, a property appears unique to the *Actinidia* family. Overall, this study reveals functional chromatin domains in plants and provides evidence for the role of three‐dimensional chromatin architecture in gene regulation and genome evolution.

## Graphical abstract

A collection of Hi‐C matrices across the plant kingdom confirms the interrelation between large‐scale chromosome conformation and species genome size. At a finer level, tandem duplicate gene clusters preferentially fold into topologically associating domains (TADs) in an evolutionarily conserved manner. Genes within TADs exhibit a higher duplication frequency, suggesting a role for TADs in promoting the origination of tandem duplicates.

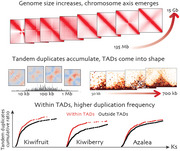

## 1 Introduction

The organization of interphase chromosomes is generally divided into two primary layers: chromosome territories and chromatin compartments. At the whole‐chromosome scale, condensin II compacts individual chromosomes, prevents centromere clustering, and defines chromosome territories.^[^ ^1^, ^2^, ^3^, ^4^ ^]^ In species lacking condensin II subunits, chromosomes adopt the Rabl configuration, where centromeres are clustered and chromosome arms are aligned in parallel.^[^ ^5^ ^]^ Within each chromosome, genomic regions are spatially segregated into distinct compartments through phase separation.^[^ ^6^, ^7^ ^]^ In some cases, these dynamic compartments are enriched with specific histone post‐translational modifications.^[^ ^8^ ^]^ A notable example is the chromocenter in *Arabidopsis*, which is established through multivalent interactions between ADCP1 and chromatin exhibiting H3K9 methylation.^[^ ^9^ ^]^ In vertebrates, an additional layer of chromatin organization is formed by the cohesin complex.^[^ ^10^, ^11^ ^]^ The NIPBL‐MAU2 loader complex loads cohesin onto DNA, and associates with it to promote loop extrusion.^[^ ^12^ ^]^ The cohesin walking on DNA is halted when it encounters CTCF proteins bound to DNA, which takes chromatin topologically associating domains (TADs) into shape.^[^ ^13^, ^14^ ^]^ The ATP‐dependent loop extrusion counteracts intrinsic chromatin compartmentalization, allowing for fine tuning of cis‐elements.^[^ ^15^ ^]^ Disruption of TAD leads to ectopic gene activation, as commonly seen in tumors.^[^ ^16^, ^17^, ^18^ ^]^

The mechanisms described above do not fully apply to plants. Several crop species, such as wheat and maize, contain all condensin II subunits but still exhibit Rabl‐like features.^[^ ^5^, ^19^, ^20^ ^]^ Intriguingly, the Rabl configuration is more frequently observed in species with large genome sizes, as emphasized in the review.^[^ ^21^ ^]^ Moreover, canonical TADs have not been identified in plant Hi‐C matrices. Although cohesin, highly conserved among eukaryotes, is essential for entrapping sister chromatids during replication,^[^ ^13^, ^22^, ^23^ ^]^ there is limited evidence for its role in altering DNA topology during interphase in plants.^[^ ^24^, ^25^ ^]^ Another key component of loop extrusion, CTCF, is absent in plants, and its functional alternatives have not been identified.^[^ ^26^ ^]^ Consequently, there remains uncertainty concerning whether TADs exist in the plant kingdom.

Notwithstanding, higher‐order chromatin organization contributes to gene regulation through various mechanisms other than loop extrusion. In *Drosophila*, GAF proteins foster long‐range promoter‐promoter interactions through their oligomerization domains, the loss of which leads to significant reductions in gene expression.^[^ ^27^ ^]^ In plants, the involvement of chromatin looping in transcriptional regulation was first identified in maize.^[^ ^28^, ^29^ ^]^ More recent work in wheat revealed a repressive loop linking H3K27me3‐labeled chromatin to the *TaTMT3B* promoter, thereby suppressing *TaTMT3B* transcription.^[^ ^30^ ^]^ In addition to the regulation of distantly separated genes and cis‐regulatory elements through looping, chromatin structure influences the expression of closely clustered genes by folding into topological domains. For instance, the activation of the avenacin biosynthetic gene cluster (BGC) in oat has been associated with chromatin domain decondensation and spatial relocation within the nucleus.^[^ ^31^ ^]^ Similar phenomena have been observed in other species^[^ ^32^ ^]^ and even in microorganisms,^[^ ^33^ ^]^ suggesting that the physical insulation of BGCs within 3D genomic space represents an ancient co‐opted mechanism for regulating BGC expression. Investigations of chromatin organization constitute a valuable approach to elucidating complex gene regulatory networks, and will shed lights on higher‐order architecture design of synthetic genomes.^[^ ^34^, ^35^ ^]^

To explore the principles of genome folding and its biological functions in plants, we constructed and analyzed Hi‐C matrices from species with diverse genome sizes and phylogenetic backgrounds. Our findings revealed that the presence of either Rabl configuration or chromosome territories was correlated with genome size, and accompanied with finer details regarding distinct patterns of compartmentalization. More interestingly, tandem duplicate gene clusters (TDGCs) defined a novel type of chromatin domain distinct from known sub‐compartments. These domains contributed to gene co‐regulation and the formation of new genes, providing insights into the role of higher‐order chromatin structure in plant genome function.

## 2 Results

### 2.1 Longer Chromosomes Exhibit Rabl Conformation and Compartment Patterns Dominated by Gene Clustering

**Figure 1:**
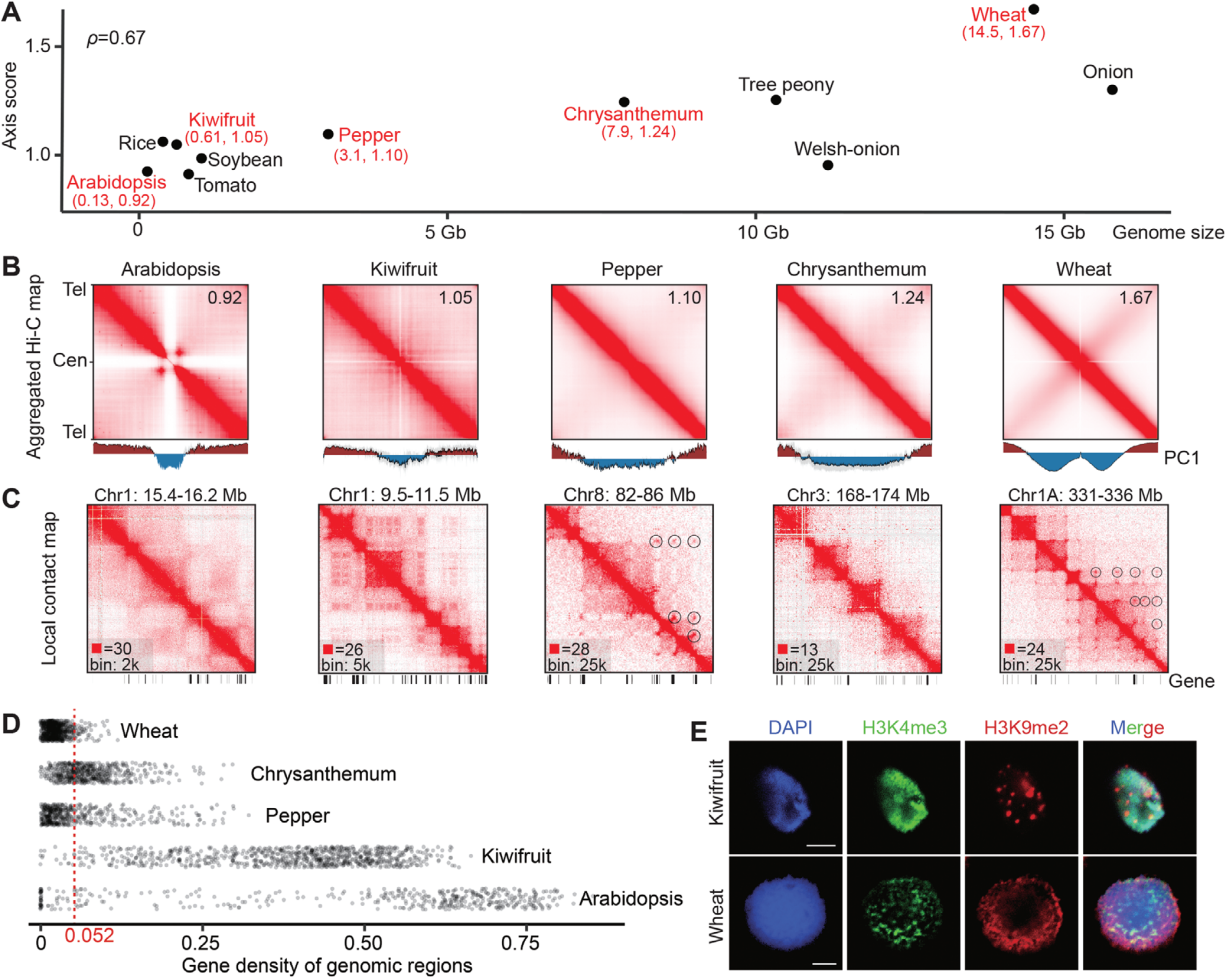
Chromatin organization in plants is correlated with genome size.A) Contact intensity along the chromosome axis in relation to genome size. Spearman rank: 0.67. Five species (colored red) were further analyzed; their coordinates are provided in brackets. B) Aggregated chromosome analysis for species with varying genome sizes. Axis scores are displayed in the top‐right corner. PC1 values for each chromosome are scaled; mean values and 95% confidence intervals are plotted. C) Chromatin organization at a finer resolution, with gene‐to‐gene loops circled for emphasis. D) Dots represent genomic blocks, and the horizontal axis indicates the gene density within each block. E) Immunofluorescence images of H3K4me3 (green) and H3K9me2 (red) in nuclei isolated from wheat and kiwifruit leaves. Scale bar: 5 µm.

We performed in situ Hi‐C on kiwifruit (*Actinidia chinensis*), a species with median genome size and well‐annotated genome we assembled previously,^[^ ^36^ ^]^ and collected Hi‐C data from species with genome sizes ranging from 135 Mb to 15.8 Gb (Table S1, Supporting Information). To characterize chromosome‐scale folding features in each species, we conducted aggregated chromosome analysis^[^ ^5^ ^]^ (ACA) and calculated contact intensity along the telomere‐to‐centromere axis (Figure S1, Supporting Information). The results revealed stronger interactions in the anti‐diagonal direction among species with larger genome sizes (r = 0.67, **Figure** **1**A). Notably, the centromeres in wheat displayed a relatively permissive nuclear environment (Figure 1B). This might be attributed to the polarized distribution of centromeres away from the main chromosome arms, characteristic of the Rabl configuration.^[^ ^19^, ^37^ ^]^ All examined species contained a complete set of condensin II subunits (Table S2, Supporting Information), for which we speculated that differences in chromosome conformation among these species were primarily attributable to variations in genome size.

We then compared the chromatin organization across species at a finer level. Typically, intergenic regions tended to form compartmental domains in gene‐sparse areas (Figure 1C, Figure S5, Supporting Information). However, the inter‐domain organization diverged into two distinct patterns. In species with small genome sizes, such as kiwifruit, the domains strode over flanking gene islands and clustered into a repressive hub (Figure S6, Supporting Information). This process aligned with the principle of compartmentalization that chromatin with similar biochemical properties tend to aggregate while dissimilar ones tend to segregate.^[^ ^7^ ^]^ Whereas in species with larger genomes, such as pepper^[^ ^38^ ^]^ and wheat,^[^ ^39^ ^]^ the most prominent compartmental contacts were established between genes rather than between intergenic regions (Figure S7, Supporting Information). These contact arrays are known as gene‐to‐gene loops (GGLs).^[^ ^39^ ^]^ To systematically evaluate GGL prevalence, we divided each genome into blocks of equal length and manually annotated the presence of GGLs within. In wheat, GGLs emerged when gene density fell below 0.052 (Figure S7B, Supporting Information), indicating that 94% of wheat genome was covered by GGL arrays (Figure 1D). In contrast, the kiwifruit genome lacked GGL structures, even in regions with gene densities below 0.052 (Figure S7C, Supporting Information). In brief, gene clustering was prevalent in wheat but absent in kiwifruit. We expected that these differences in chromatin organization could be observable under a microscope.

Immunolocalization of transcriptionally active euchromatin (carrying H3K4me3) and heterochromatin (carrying H3K9me2) confirmed distinct nuclear organization patterns in kiwifruit and wheat. In kiwifruit, heterochromatin appeared highly condensed (Figure 1E), similar to the chromocenter structures observed in *Arabidopsis*.^[^ ^40^ ^]^ Conversely, wheat displayed a more diffuse heterochromatin distribution within the nucleus. Moreover, fluorescent signals corresponding to H3K4me3 were localized in discrete foci, supporting the hypothesis that genes connected by consecutive loops cluster together to form transcription factories.^[^ ^39^ ^]^ In summary, these findings indicated that a species’ genome size influences large‐scale chromosome conformation as well as finer details of chromatin compartmentalization.

### 2.2 Tandem Duplicate Gene Clusters Preferentially Fold into TAD‐Like Domains in Plant Genome

**Figure 2:**
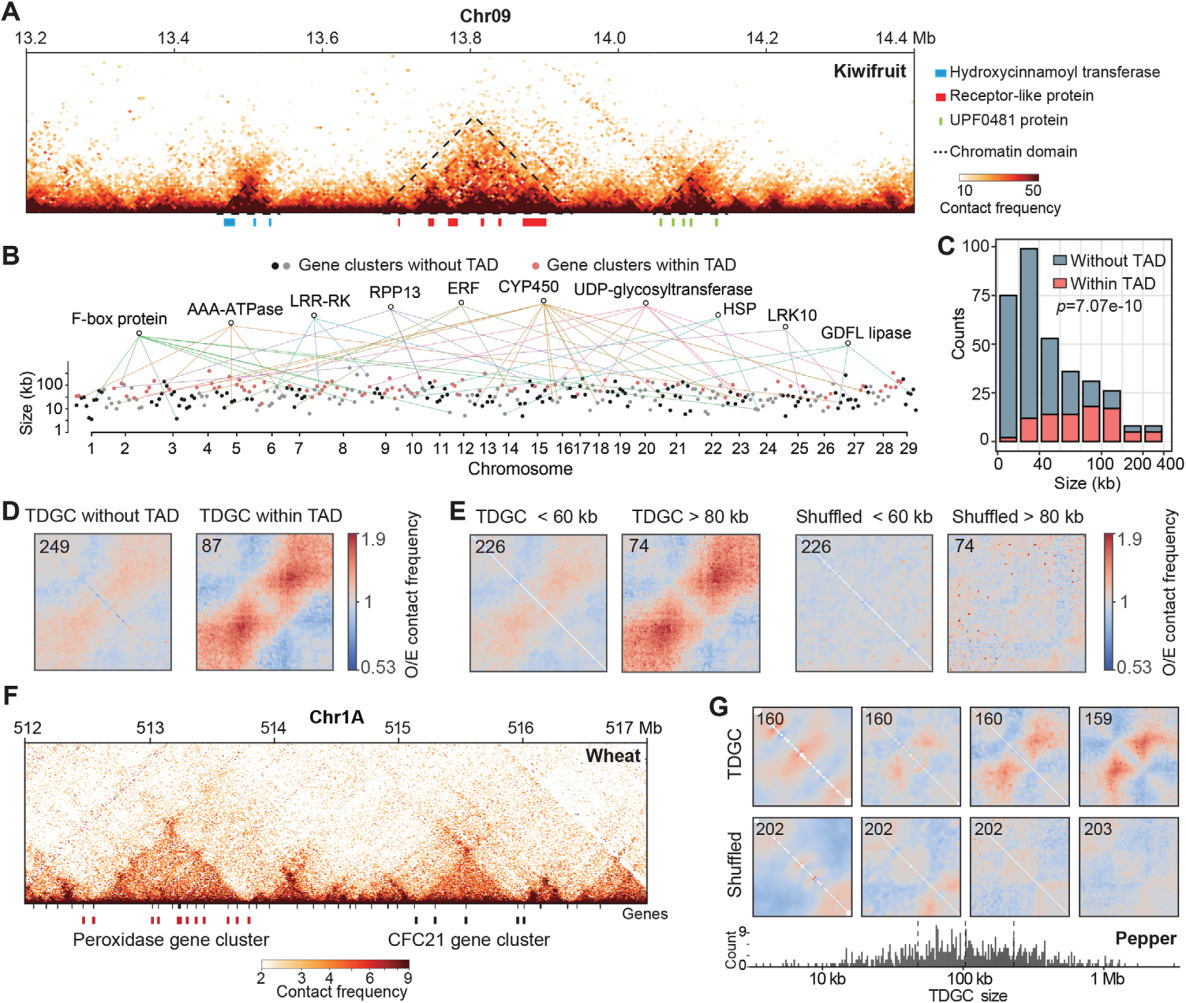
Organization of tandem duplicate gene clusters (TDGCs) into TAD‐like domains across multiple species.A) Representative Hi‐C heatmap from kiwifruit showing the colocalization of tandem duplicate gene clusters with chromatin domains identified by Arrowhead. B) Each dot represents a TDGC; those located within TAD‐like domains are colored red. The horizontal axis shows the genomic positions of gene clusters, and the vertical axis indicates their spanning lengths. C) Numbers of TDGCs within and outside TAD‐like domains as a function of spanning length. D) Validation of TDGCs within and outside TAD‐like domains using pile‐up analysis. The number of piled TDGCs is shown in the top‐left corner. Matrix resolution: 2 kb. Rescaled length: 100 kb. E) Pile‐up analysis comparing TDGCs with small and large spanning lengths. F) Representative Hi‐C heatmap from wheat illustrating the colocalization of TDGCs with chromatin domains. G) Local chromatin organization of TDGCs in pepper. TDGCs are sorted and aggregated based on their spanning lengths. Random gene arrays of similar size are used as controls.

We used Arrowhead^[^ ^11^ ^]^ to identify potential TADs, beginning with the kiwifruit genome. In total, 1250 domains were identified within euchromatin regions, with an average length of 123.4 kb (Figure S8, Supporting Information). Notably, a subset of these domains contained large clusters of multi‐copied genes, namely tandem duplicate gene cluster (TDGC) (**Figures** **2**A, and S9, Supporting Information). To investigate the relationship between chromatin domains and tandem duplicates, we identified 336 TDGCs across the kiwifruit genome, each containing at least three copies of the same gene (Figure 2B). Upon comparison of TDGC locations using TAD coordinates inferred by Arrowhead, we found that 62% of TDGCs larger than 80 kb were embedded within chromatin domains. For TDGCs smaller than 60 kb, this proportion decreased to 14% (Figure 2C). Overall, tandem duplicate genes were preferentially located within TAD‐like domains compared to random condition (at least threefold difference, *p* = 7.07e‐10 by hypergeometric test) (Figure 2D,E), and such preference was found to be conserved among different species (Figure S10, Supporting Information). In organisms with large genomes (e.g., pepper and wheat), where chromatin often folds into continuous contact domains (Figures 2F, and S5, Supporting Information), colocalization with TAD‐like domains remained stronger for TDGCs than for random gene arrays (Figure 2G, see Methods). These findings suggest that the preferential organization of tandem duplicates into self‐contacting domains is a universal feature of plant genomes.

### 2.3 The Structure of TDGC‐Embedded TAD‐Like Domain is Independent of Chromatin Compartmentalization and Conserved Among Species

**Figure 3:**
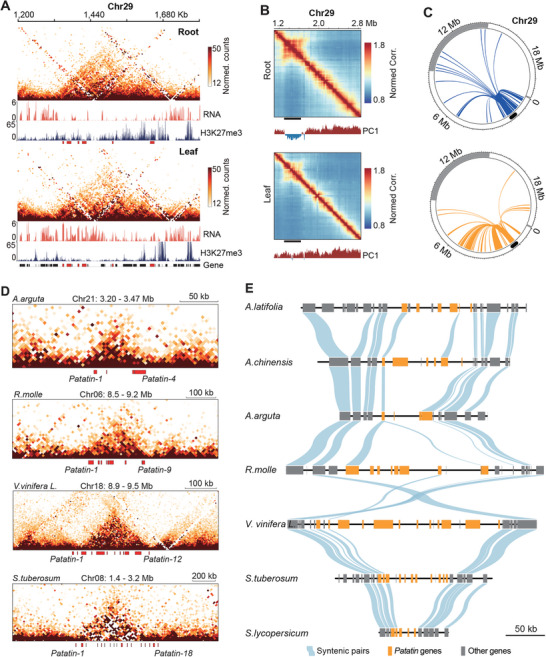
TAD‐like domains encompassing TDGCs are independent of compartments and evolutionarily conserved across species.A) TAD‐like domain encompassing the terpineol synthase (*TPS*) gene cluster. *TPS* genes are colored red. Hi‐C matrices have been normalized to the same read coverage. B) Pearson correlation matrices with PC1 values indicating repressive compartments (blue) and permissive compartments (red). C) Circos plots illustrating intrachromosomal differential genomic interactions for the *TPS* cluster in leaves and roots. Chromosome arms are shown in white, central B compartments in gray, and the *TPS* cluster in black. D) Chromatin organization of syntenic *patatin* gene clusters in *A. arguta*, *R. molle*, and *V. vinifera*, along with a non‐syntenic *patatin* gene cluster in potato. E) Microsyntenic segments centered on *patatin* genes. All segments are scaled to the same length.

TAD‐like domains encompassing TDGCs were distinct from compartmental domains or loop domains formed through loop extrusion. First, they lacked the punctate interaction signals at domain corners that typically indicate loop anchors. Second, these TAD‐like domains were independent of chromatin states. Whereas H3K27me3 deposition contributed to domain condensation and mediated compartmental switches at domain loci, the loss of H3K27me3 had minimal impact on the structural integrity of TAD‐like domains (Figure S11, Supporting Information). A representative example is the terpineol synthase (*TPS*) gene cluster. In kiwifruit leaf tissue, the *TPS* domain existed in the complete absence of H3K27me3 (**Figure** **3**A). Compared with root tissue, this domain underwent a dramatic spatial relocation from the B compartment to the A compartment (Figure 3B) and displayed increased interactions with neighboring euchromatin regions (Figure 3C). Despite these changes, the fundamental domain architecture remained intact, suggesting that domains formed by TDGCs are highly stable and can persist independently of chromatin compartmentalization. Moreover, we checked the chromatin domain formation on another 338 co‐expressing gene arrays that were not tandem duplicates, in order to investigate if these TAD‐like domains were resultant from co‐expressing compartment (see Methods). No higher‐order chromatin architecture was detected (Figure S12, Supporting Information). Based on these findings, we propose that tandem duplicate gene clusters could define a novel type of TAD‐like domain in kiwifruit genome.

Next, we explored the conservation of TAD‐like domains across species, using the *patatin* gene cluster as an example. In kiwifruit, six *patatin* genes were closely arranged within a ≈114‐kb genomic region, which folded into a distinct chromatin domain (Figure S13, Supporting Information). The syntenic *patatin* cluster in *Actinidia arguta*, *Actinidia latifolia* and their close relatives, *Rhododendron molle* and *Vitis vinifera* all folded into a chromatin domain, regardless of variations in gene copy number (Figure 3D,E). In potato, although we were unable to identify the syntenic *patatin*‐embedded domain due to the sparse Hi‐C matrix,^[^ ^41^ ^]^ an 18‐copy *patatin* cluster spanning ≈700 kb on chromosome 8 was perfectly colocalized with a defined TAD‐like domain as well. The only exception was tomato, where the *patatin* cluster (≈33 kb) was too small to form a chromatin domain (Figure S13, Supporting Information). Overall, 21 of 53 syntenic TDGC‐embedded TAD‐like domains were conserved between *A. chinensis* and *A. arguta*, and this number was 14/35 for syntenic TAD‐like domains between *A. chinensis* and *R. molle*. These results suggest that the structure of TAD‐like domains encompassing TDGCs is relatively conserved among species.

### 2.4 TAD‐Like Domains Encompassing TDGCs Play a Limited Role in Gene Co‐Regulation in Actinidia Chinensis

**Figure 4:**
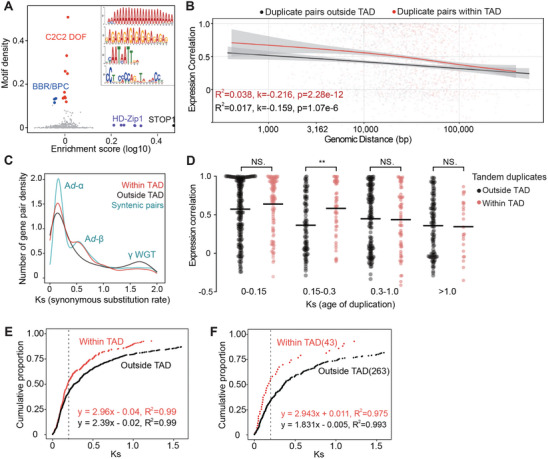
Duplicate pairs within TAD‐like domains are younger and more coregulated in *Actinidia chinensis*.A) Motif enrichment analysis of TDGC sequences. Fold enrichment is calculated as the ratio of motif density within TDGCs relative to that in the 40‐kb flanking genomic regions. Motif density is measured in 1‐kb genomic bins. Position weight matrices (from top to bottom) correspond to DOF, BPC, HD‐Zip1, and STOP1, respectively. B) Scatter plot showing the relationship between expression correlation and genomic separation for duplicate pairs inside and outside TAD‐like domains. C) Distribution of synonymous substitution rates (Ks values) for duplicate pairs inside and outside TAD‐like domains. Collinear paralogs identified across the *A. chinensis* genome are shown in light blue. D) Expression correlation of tandem duplicates inside and outside TAD‐like domains. Asterisks (**) indicate significant differences (*p* = 1.2e‐3, Wilcoxon test). E) Cumulative distribution of all tandem duplicates in *A. chinensis* as a function of Ks. F) Cumulative distribution of tandem duplicates within three‐copy TDGCs in *A. chinensis* as a function of Ks. Numbers in brackets indicate the sample size.

We explored the factors influencing the formation of TAD‐like domains encompassing TDGCs. Hypothesizing that a certain type of protein bind to DNA and promote DNA conformational change, we measured the motif abundance of over 400 transcription factors^[^ ^42^ ^]^ in the TDGC sequences of kiwifruit. Results revealed that the STOP1 protein from the C2H2 family ranked highest in abundance while BPC proteins were rather depleted (**Figure** **4**A), the latter of which have been shown to be capable of structuring a chromatin domain.^[^ ^43^, ^44^ ^]^ However, we questioned the role of STOP1 in domain formation because its motifs also were abundant in TDGCs outside of TAD‐like domains (Figure S15A, Supporting Information). We extended this analysis to other species. However, completely new sets of TFs were enriched while STOP1 was absent (Figure S15B, Supporting Information). This variability prevented us from identifying a specific protein responsible for shaping TAD‐like domains; or alternative mechanisms might be involved in their formation.

Concerning the functional role of TAD‐like domains, we speculated that the domain structure might facilitate the co‐regulation of linearly distant genes by bringing them into proximity, allowing shared cis‐ or trans‐regulatory elements to act more effectively.^[^ ^45^ ^]^ To test this hypothesis, we controlled for factors influencing co‐expression levels of duplicate genes, including genomic distance and duplication age.^[^ ^46^ ^]^ We first calculated the genomic distances between duplicate pairs located inside and outside TAD‐like domains, then inferred their expression correlations using data from 45 kiwifruit RNA sequencing datasets.^[^ ^36^ ^]^ The results showed that expression correlation decreased monotonically with increasing genomic distance (*p* < 1.07e‐6, F‐test). However, duplicate pairs within TAD‐like domains exhibited significantly higher expression correlations relative to those outside the domains (Figure 4B). This finding suggested that TAD‐like domains might contribute to gene co‐regulation. Next, we took duplication age into consideration given the fact that transcription divergence between duplicates accumulates over evolutionary time.^[^ ^46^ ^]^ The synonymous substitution rate (Ks) was used as a proxy for duplication age. We observed that duplicate pairs inside and outside TAD‐like domain exhibited distinct Ks distribution (Figure 4C): duplicate pairs within domains had lower Ks values, predominantly originating during two whole‐genome duplication events^[^ ^47^, ^48^ ^]^ (Ks = 0.15 and Ks = 0.5). Conversely, duplicate pairs outside domains also extensively originated during the ancient triplication event shared by core eudicots^[^ ^49^, ^50^ ^]^ (Ks = 1.7). We thus controlled for both genomic distance and duplication age (Figure S16, Supporting Information), and observed that the co‐regulation effect attributed to TAD‐like domain structure largely disappeared. Only a small subset of genes (Ks = 0.15–0.3) exhibited significantly different expression correlations between inside and outside TAD‐like domains (Figure 4D). These results suggest that the role of TAD‐like domains in gene co‐regulation is limited or varies across evolutionary times (Figure S17, Supporting Information).

### 2.5 TAD‐Like Domains Potentially Facilitate the Formation of Tandem Duplicates in Actinidia Chinensis

Considering that TAD‐like domains harbored duplicate pairs with more recent divergence times, we hypothesized that these domains might play a role in tandem duplicate origination in kiwifruit. Accordingly, we estimated duplication frequency based on the abundance of the very youngest tandem duplicates.^[^ ^51^, ^52^ ^]^ In kiwifruit, there were 24 of 243 tandem duplicates embedded in the domain with Ks < 0.05. In contrast, for tandem duplicates outside TAD‐like domains, this number was a quarter lower (38/497, Figure S18, Supporting Information). We further computed the cumulative distribution of tandem duplicates as a function of Ks, and observed that tandem duplicates accumulated more rapidly within TAD‐like domains (Figure 4E). To estimate the absolute birth rate of tandem duplicate, we applied a linear model to the curve with Ks ≤ 0.2, considering a minimal saturation effect.^[^ ^53^ ^]^ The analysis revealed a slope of 2.96 for genes within TAD‐like domains, corresponding to a duplication rate of ≈0.01664 new duplicates per gene per million years (assuming a synonymous substitution rate of 2.81e‐9 mutations per site per year^[^ ^54^ ^]^). In contrast, the duplication rate for genes outside TAD‐like domains was 0.01343 per gene per million years. These results suggest that TAD‐like domains associate with the rapid formation of tandem duplicates in the kiwifruit genome.

However, we needed to determine whether this difference in duplication frequency was influenced by variations in gene cluster size, as larger TDGCs are more susceptible to gene conversion,^[^ ^53^ ^]^ a process where homologous DNA sequence information from one fragment is transferred to another during recombination, thereby reducing nucleotide divergence between older duplicates.^[^ ^55^ ^]^ In kiwifruit, the average length of TDGCs within TAD‐like domains was 77.6 kb, substantially larger than the 31.4 kb average length for TDGCs outside these domains (Figure S19, Supporting Information). Additionally, the number of gene copies per cluster considerably differed: TDGCs within and outside TAD‐like domains contained an average of 5.48 and 3.92 genes, respectively. To mitigate the effect of gene conversion, we restricted our analysis to three‐copy gene clusters. A total of 306 pairs of tandem duplicates were identified within three‐copy TDGCs for further analysis. We obtained the Ks values for these tandem duplicates and generated a cumulative distribution plot. The results showed that even with the same gene cluster size, genes within TAD‐like domains exhibited a significantly higher duplication frequency (a 1.6‐fold increase, Figure 4F). These findings confirm that the impact of TAD‐like domains on the birth rate of tandem duplicates is real, and not simply a bias introduced by differences in TDGC size.

### 2.6 TAD‐Like Domains do not Contribute to Duplicate Origination in Rhododendron

**Figure 5:**
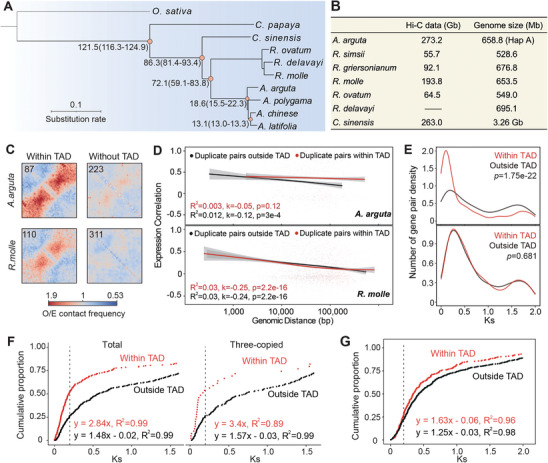
TAD‐like domains potentially facilitate the formation of tandem duplicates in the *Actinidia* family.A) Phylogenetic tree of 10 species, with divergence times labeled at each node. B) Table summarizing available Hi‐C data volumes and genome sizes for close relatives of kiwifruit. C) Identified TDGCs within and outside TAD‐like domains in *A. arguta* and *R. molle*. The total number of piled intervals is shown in the top‐left corner. D) Co‐expression levels for duplicate pairs inside and outside TAD‐like domains in *A. arguta* and *R. molle*, plotted as a function of genomic distance. E) Distribution of synonymous substitution rates (Ks values) for duplicate pairs inside and outside TAD‐like domains in *A. arguta* and *R. molle*. *P*‐values, derived from t‐tests with Bonferroni correction, indicate statistical differences between the two curves. F) Cumulative distribution of all tandem duplicates and those within three‐copy TDGCs in *A. arguta* as a function of Ks. G) Cumulative distribution of all tandem duplicates in *R. molle*.

To investigate whether TAD‐like domains influence duplicate origination in other species, we collected Hi‐C and RNA‐seq data from close relatives of kiwifruit. The genus *Rhododendron* (Ericaceae), which includes more than 1000 species, diverged from *Actinidia* ≈72 million years ago (Mya)^[^ ^47^, ^56^ ^]^ (**Figure** **5**A). We selected *R. molle* for analysis due to its comparable genome size, gapless genome assembly, and the availability of recently published high‐quality Hi‐C data^[^ ^57^ ^]^ (Figure 5B). Within Actinidiaceae, *Actinidia arguta* was the first to diverge (≈18.6 Mya) among all sequenced species^[^ ^47^ ^]^ and was chosen for further analysis.

In total, 310 and 421 TDGCs were identified in *A. arguta* and *R. molle* using the pipeline described before. Because Arrowhead could not reliably detect chromatin domains in low‐resolution Hi‐C matrices, we tested whether a TDGC formed a TAD‐like domain by calculating its inter/intra contact ratio (see Methods). Manual inspection of TDGCs revealed that 28.1% in *A. arguta* and 26.1% in *R. molle* were embedded within defined TAD‐like domains (Figure 5C), comparable to the percentage observed in kiwifruit (25.9%).

We then calculated the expression correlation and synonymous divergence (Ks) for every combination of duplicate pairs in TDGCs within and outside TAD‐like domains. In *A. arguta*, the domain structure effectively mitigated the distance‐dependent decay in gene co‐expression (Figure 5D) and contained duplicate pairs with much more recent divergence times (Figure 5E). Conversely, in *R. molle*, duplicate pairs inside and outside TAD‐like domains exhibited similar levels of co‐expression and nearly identical Ks distributions (Student's t‐test, *p* = 0.681). These findings imply that the roles of TAD‐like domains in gene regulation and genome evolution are species‐specific.

Subsequently, we estimated the rates of tandem duplicate formation using their cumulative distributions. In *A. arguta*, tandem duplication occurred at a rate of ≈0.01596 per gene per million years within TAD‐like domains, compared with 0.0083 for tandem duplicates outside chromatin domains (Figure 5F). The difference was even more pronounced in three‐copy TDGCs, where over half of the tandem duplicates within TAD‐like domains exhibited Ks ≤ 0.2. Considering these results in conjunction with findings from *A. chinensis*, we proposed that TAD‐like domains might facilitate the formation of tandem duplicates in the *Actinidia* genome.

We conducted the same analysis on *R. molle* and found that tandem duplications accumulated at a slightly faster rate within TAD‐like domains (Figure 5G). However, after controlling for gene cluster size, this difference was no longer significant (Figure S20D, Supporting Information). Therefrom, the TAD‐like domain structure likely less associates with the emergence of duplicates in *Rhododendron*.

## 3 Discussion

In 1979, researchers systematically studied the nuclear ultrastructure of 15 angiosperms and concluded that nuclear type was primarily determined by nuclear DNA content.^[^ ^58^ ^]^ Since then, various immunostaining studies have further established the relationship between chromatin organization and genome size.^[^ ^59^, ^60^ ^]^ In the present study, by integrating Hi‐C matrices from species with varying genome sizes, we found that differences in chromatin organization could be characterized by the presence of GGLs.

GGL arrays are presumably associated with RNA polymerase II (Pol II).^[^ ^39^ ^]^ In living cells, Pol II forms clusters at active genes through multivalent interactions between its carboxy‐terminal domain and mediator complexes.^[^ ^61^, ^62^ ^]^ These transcription‐centered compartments facilitate the localized deployment of regulatory factors to a limited number of “focal points,” a mechanism particularly important for organisms with large intergenic regions, such as wheat^[^ ^39^ ^]^ and pepper.^[^ ^38^ ^]^ However, no GGL arrays were observed in gene‐sparse regions of species such as kiwifruit, rice, or *Arabidopsis*, even when genes in these regions were actively transcribed (Figure S16, Supporting Information). These findings suggest that the formation of GGL structures is not solely driven by transcriptional machinery but may also depend on the overall genome composition, including the heterochromatin‐to‐euchromatin ratio.

Several studies have demonstrated that volume exclusion occurs in nuclear compartments, where the space occupied by co‐solutes becomes inaccessible to other proteins.^[^ ^63^ ^]^ According to this principle, large volumes of heterochromatin inevitably displace euchromatin and its associated binding proteins, leading to the spatial aggregation of Pol II and H3K4me3‐labeled euchromatin, or vice versa. Similarly, enforced molecular crowding has been shown to induce large‐scale chromatin compaction and segregation of other nuclear components in human cells.^[^ ^64^ ^]^ Therefore, we speculate that heterochromatin exclusion and Pol II‐mediated clustering function collaboratively to form GGL arrays. This hypothesis could be further validated through polymer simulations.^[^ ^65^ ^]^

Another key finding of our study is the preferential organization of TDGCs into TAD‐like domains. This preference is evolutionarily conserved across species and is more pronounced in larger TDGCs.

Our findings enable an intriguing comparison to previous reports. First, TAD‐like domains encompassing TDGCs in our study were independent of chromatin compartments and were highly conserved across tissues. In contrast, *Arabidopsis* domains containing biosynthetic gene clusters exhibited tissue‐specific conformations closely linked to transcriptional state and H3K27me3 deposition. Moreover, whereas TADs in *Marchantia* were frequently associated with TCP1 proteins,^[^ ^26^ ^]^ the interiors of TDGCs in *A. chinensis* were enriched with binding sites for STOP1, a Cys(2)His(2)‐type zinc finger protein. In pepper, *A. arguta*, and *R. molle*, however, entirely different sets of TFs such as MYB70 and CAMTA5 were found to be enriched in TDGCs. These TFs were involved in responses to environmental stimuli like ethylene,^[^ ^66^ ^]^ cold,^[^ ^67^ ^]^ and phosphate starvation.^[^ ^68^ ^]^ Considering that tandem duplications are more likely to occur in genes involved in environmental responses, we suspect that this preference underlies the observed enrichment of these TFs in TDGCs.

One unexpected finding was that genes within TAD‐like domains exhibited a higher frequency of tandem duplication. Given that tandem duplication arises from unequal crossovers,^[^ ^69^ ^]^ the TAD‐like domain structure may play a role in patterning recombination frequency during meiosis.^[^ ^70^, ^71^ ^]^ Similar observations have been made in the rice genome, where TADs show increased single‐nucleotide polymorphism and structural variation density, along with higher recombination rates.^[^ ^72^ ^]^ However, in *R. molle*, such functionalization of TAD‐like domains was not observed. This absence might be attributed to the relatively low duplication frequency in the *Rhododendron* genome (Figure S20, Supporting Information), reducing the opportunity for chromatin domains to exert widespread effects. In this respect, the variation in crossover rates among species might explain why the role of TAD‐like domains in genome evolution is species‐specific.^[^ ^73^ ^]^ Nevertheless, much exploration remains necessary regarding how interphase chromatin structure influences chromatin behavior during meiosis, in which chromatin undergoes substantial linear and axial compaction that potentially disrupts local topological organization.^[^ ^74^, ^75^ ^]^

## 4 Conclusion

By integrating Hi‐C data from species with varying genome sizes and phylogenetic backgrounds, we identified several key findings. First, genome size influences large‐scale chromosome conformation and detailed compartmental patterns, which are characterized by the presence of GGLs. Second, we identified a novel type of chromatin domain composed of TDGCs. These TAD‐like domains are evolutionarily conserved and distinct from traditional compartmental or loop domains. Third, TAD‐like domains may contribute to the formation of tandem duplicates in the *Actinidia* genome. These findings provide new insights into the role of higher‐order chromatin structures in genome functionalization and evolution.

## 5 Experimental Section

### Plant Materials

Female *Actinidia chinensis* var. “Donghong” plants were grown at Wuhan Botanical Garden, Chinese Academy of Sciences (30.5° N, 114.4° E). Fruits were collected at the early stage of fruit development on May 24, 2022. Young leaves were harvested on May 27, 2022; branch roots were sampled from 1‐year‐old saplings on June 24, 2022. Six samples (two biological replicates per tissue) were immediately frozen in liquid nitrogen and stored at −80 °C. The wheat cultivar Chinese Spring was cultivated in a growth chamber under a 16‐h photoperiod, with daytime and nighttime temperatures maintained at 22 and 17 °C, respectively.

### Hi‐C Library Construction and Sequencing

Hi‐C libraries were constructed and sequenced by Frasergen Bioinformatics Co., Ltd. (Wuhan, China). Plant samples were ground into powder and cross‐linked in 2% formaldehyde solution for 15 min. After cross‐linking and quenching, the fixed nuclei were subjected to *Mbo*I digestion. The digested DNA was end‐labeled with biotin‐14‐dCTP. Proximal DNA ends were re‐ligated, purified, and sonicated into 250‐ to 400‐bp fragments. The DNA fragments were subjected to blunt‐end repair, A‐tailing, and adaptor ligation. Streptavidin C1 beads were used to pull down the biotin‐labeled DNA, which was then amplified by polymerase chain reaction. The amplified libraries were sequenced on the DNBSEQ platform.

### Hi‐C Data Processing

Paired‐end raw sequence reads were trimmed using fastp (version 0.23.2)^[^ ^76^ ^]^ and mapped to the *A. chinensis* var. “Donghong” genome using the HiC‐Pro pipeline.^[^ ^77^ ^]^ To assess the reproducibility of each pair of replicates, contact matrices were evaluated using HiCRep^[^ ^78^ ^]^ at a resolution of 20 kb. After confirming data quality, replicates were merged into a single .validPairs file, which was subsequently converted to a .hic file with KR normalization. Visualization of .hic files at various resolutions was performed using Juicebox.^[^ ^79^ ^]^ Hi‐C matrices from different tissue samples were normalized to the same read coverage using hicNormalize^[^ ^80^ ^]^ prior to further comparisons. To obtain Hi‐C matrices from published studies, raw sequencing reads were downloaded from the NCBI Sequence Read Archive and the CNCB Genome Sequence Archive (Table S3, Supporting Information) and mapped to the latest versions of the respective genome assemblies.^[^ ^36^, ^38^, ^41^, ^47^, ^57^, ^81^, ^82^, ^83^, ^84^, ^85^, ^86^, ^87^, ^88^, ^89^ ^]^

### Aggregated Chromosome Analysis

The sorted and deduplicated .validPairs files were used to perform ACA with the script provided in the 3D‐DNA release (supp/build‐aca‐hic.sh).^[^ ^90^ ^]^ The aggregated Hi‐C matrix was binned with a bin size equal to 1/20 of the virtual chromosome length. KR‐normalized contact counts from the first six bins in the anti‐diagonal direction were summed and divided by the counts from the first six bins on the right side. This ratio, referred to as the axis score, represented contact intensity along the telomere‐to‐centromere axis. To enable alignment with the aggregated Hi‐C matrix, PC1 values for each chromosome were scaled using an in‐house script. Mean PC1 values with 95% confidence intervals were plotted. Additional details are provided in the supplementary file.

### Immunofluorescence

Young leaves from kiwifruit and the wheat cultivar Chinese Spring were used for immunofluorescence analysis. ≈1 g of tissue was immersed in 4 mL of NIB (nuclear isolation buffer; see CUT&Tag and data processing section) and chopped into a suspension. The suspension was filtered, and 3 mL of 4% formaldehyde were added. The mixture was gently swirled and stored at room temperature (RT) for 15 min. Fixation was stopped by adding 1 mL of 1 M glycine. The sample was subjected to two rounds of centrifugation and washing; the nuclear pellet was subsequently resuspended in 100 µL of NSB (nuclear suspension buffer; see CUT&Tag and data processing section). A 10‐µL aliquot of the nuclear suspension was dropped onto SuperFrost slides (Epredia) and air‐dried for 30 min. The slides were treated with 500 µL of 10 mM NaBH_4_ for 7 min to eliminate autofluorescence and washed three times with phosphate‐buffered saline for 5 min each. Slides were incubated with blocking buffer (phosphate‐buffered saline containing 5% bovine serum albumin and 0.1% Triton X‐100) for 30 min at RT. Primary antibodies against H3K4me3 (Millipore, CS200508) and H3K9me2 (Abcam, ab1220) were diluted 1:400 in blocking buffer. Slides were incubated with primary antibodies for 1 h at RT, then washed and incubated with secondary antibodies diluted 1:400 in blocking buffer. Alexa Fluor 488 (Abcam, Ab150077) was used to label H3K4me3, and Alexa Fluor 647 (Abcam, Ab150115) was used to label H3K9me2. After incubation for 1 h in the dark, slides were mounted with antifade mounting medium containing 4′,6‐diamidino‐2‐phenylindole (DAPI; Vectashield) and observed using a NIKON A1 confocal microscope through a ×60 oil‐immersion objective. Raw images were processed using NIS Elements Viewer software.

### Identification of TDGCs within TAD‐Like Domains

Tandem duplicates were identified using MCScanX.^[^ ^50^ ^]^ TDGCs were defined as clusters of consecutive tandem duplicates with a gene copy number ≥3 and no more than four intervening non‐homologous genes. Colocalization of TDGCs with TAD‐like domains was determined using their inter/intra contact ratio^[^ ^89^ ^]^ on Hi‐C matrices. Specifically, the inter‐contact intensity between the 40‐kb genomic block adjacent to the TDGC and the TDGC itself was calculated, then divided by the intra‐contact intensity within the TDGC region. Lower ratios indicated more prominent chromatin domains in the TDGC region. For *A. chinensis*, *A. arguta*, and *R. molle*, Hi‐C matrix resolutions of 5, 5, and 10 kb were used, respectively.

### Identification of Co‐Expressing Gene Arrays

In total, 40 977 genes were filtered using the goodSamplesGenes function in the R package “WGCNA.”^[^ ^91^ ^]^ The resulting 39 024 genes were grouped into 67 co‐expressing modules based on their FPKM values across 45 tissue samples, using the parameters power = 7, minModuleSize = 30, and deepSplit = 2. Co‐expressing gene arrays were defined as clusters of consecutive genes belonging to the same module, with at least three genes and no more than one intervening non‐homologous gene. Tandem duplicate genes were excluded from this analysis.

### Chromatin Compartment Identification

Chromatin compartments were identified using hicexplorer.^[^ ^80^ ^]^ The .hic file was converted to a .cool file using the hicConvertFormat tool. PC1 values for chromosome loci were calculated with hicPCA using the parameters –whichEigenvectors 1 –pearsonMatrix. The signs of PC1 values across chromosomes were corrected using the TE distribution, with negative PC1 values assigned to TE‐rich regions. Compartment allocation was robust across bin sizes ranging from 25 kb to 100 kb. Finally, Pearson matrices and feature tracks were plotted using pyGenomeTrack.^[^ ^92^ ^]^

### Long‐Range Contact Pattern Depiction

The .hic files for each replicate were converted into contact dataframes at 50‐kb resolution using straw.^[^ ^79^ ^]^ Leaf and root contact dataframes were analyzed using the R package “multiHiCcompare.”^[^ ^93^ ^]^ Differential contacting loci between tissues within a defined domain region were identified using the parameter p.adj ≤ 0.05. Circos plots were generated via the “circlize” package.^[^ ^94^ ^]^

### Pile‐Up Analysis

For a given set of genomic intervals, local contact heatmaps were aggregated using coolpup.py^[^ ^95^ ^]^ with the –rescale –local parameters and an OE matrix as input. Random intervals were generated as controls using bedtools shuffle. Heterochromatin regions with the lowest gene density were excluded from the randomization process.

### Motif Enrichment Analysis

TF motif files (position weight matrices) were downloaded from the *Arabidopsis* DAP‐seq database (http://neomorph.salk.edu/PlantCistromeDB).^[^ ^42^ ^]^ Genome‐wide motif distribution was determined using fimo^[^ ^96^ ^]^ with the parameters –thresh 1e‐5 –max‐stored‐scores 100 000 000. For each motif, the enrichment score was calculated as the ratio of motif density within TDGCs to that in the 40‐kb flanking genomic regions.

### Ks Distribution and Expression Correlation Analysis

For a TDGC containing n gene copies, the number of all possible combinations of duplicate pairs was calculated as [n(n‐1)]/2. Synonymous substitution rates (Ks values) for each gene pair were calculated using paraAT.^[^ ^97^ ^]^ To capture whole‐genome duplication events, collinear gene pairs within the *A. chinensis* genome were also identified for Ks calculation. Ks distributions were visualized using the geom_density() function in the R package “ggplot2.” Gene expression correlations were derived from RNA‐seq datasets, including 45, 30, and 24 samples for *A. chinensis*,^[^ ^36^ ^]^ *A. arguta*,^[^ ^47^ ^]^ and *R. molle*,^[^ ^57^ ^]^ respectively. Genomic distances and expression correlations were fitted using a linear model with the lm() function in R.

### Estimation of Duplication Frequency

For a TDGC containing n gene copies, the maximum number of tandem duplication events was (n‐1). Tandem duplicates were identified using MCScanX,^[^ ^50^ ^]^ and Ks values were computed via paraAT.^[^ ^97^ ^]^ The cumulative distribution of tandem duplicates as a function of Ks was plotted in R. A linear model was fitted using the lm() function, and the slope (k) of the linear function for Ks ≤ 0.2 was used to estimate the duplication rate (x/T) according to the equation: *x*/*T* = 2*kμ*, where *μ* = 2.81e‐9.^[^ ^54^ ^]^

### Phylogenetic Analysis

Syntenic genes were identified by MCScanX^[^ ^50^ ^]^ and plotted by NGenomeSyn.^[^ ^98^ ^]^ Single‐copy orthologs were identified for *A. chinensis*, *A. arguta*, *A. polygama*, *A. latifolia*, *R. molle*, *R. ovatum*, *R. delavayi*, *C. sinensis*, *C. papaya*, and *O. sativa* using OrthoFinder.^[^ ^99^ ^]^ These orthologs were aligned using MUSCLE.^[^ ^100^ ^]^ The alignments were trimmed with Gblocks (version 0.91b) prior to transformation into a .phy file. A maximum likelihood tree was constructed using RaxML^[^ ^101^ ^]^ with the following parameters: ‐f a ‐p 123 456 ‐x 123 456 ‐N 1000 ‐m PROTGAMMAJTT. The resulting phylogenetic tree was visualized with MEGA11.^[^ ^102^ ^]^ Species divergence times were estimated using mcmctree^[^ ^103^ ^]^ and calibrated using divergence data from the TimeTree^[^ ^104^ ^]^ database (http://www.timetree.org/). The Ks distributions of these single‐copy orthologs were computed using paraAT^87^ and plotted with the geom_density() function in the R package “ggplot2.”

### CUT&Tag and Data Processing

We modified the nuclear isolation protocol for kiwifruit and used the CUT&Tag kit (Vazyme #TD903) to generate a histone modification atlas. ≈2 g of tissue were immersed in 10 mL of NIB (nuclear isolation buffer: 10 mM MgSO_4_, 0.5 mM 4‐(2‐hydroxyethyl)‐1‐piperazineethanesulfonic acid [HEPES], 5 mM KCl, 1 mg mL^−1^ dithiothreitol, 0.25% Triton X‐100)^[^ ^105^ ^]^ and quickly chopped into a suspension on ice. The homogenate was filtered through a 40‐µm strainer, transferred to 2‐mL tubes, and centrifuged at 2000 × g for 5 min at 4 °C. The supernatant and green precipitates were carefully removed, and the white pellet was resuspended in 1 mL of NSB (nuclear suspension buffer: 10 mM MgSO_4_, 0.5 mM HEPES, 5 mM KCl, 1 mg mL^−1^ dithiothreitol).^[^ ^105^ ^]^ The nuclear suspension was centrifuged at 1500 × g for 5 min at 4 °C, and the supernatant was discarded. The white pellet was dissolved in 500 µL of wash buffer. To evaluate nuclei quality and count, 10 µL of the suspension were stained with DAPI. ≈100 000 nuclei were transferred to three fresh 1.5‐mL tubes: two for replicates and one for a negative control. Primary antibodies against H3K27me3 (Diagenode, C15410195) and H3K4me3 (Millipore, CS200508) were used at a 1:50 dilution. Goat anti‐rabbit IgG (1:100 dilution) served as the secondary antibody. The libraries were amplified in 15 polymerase chain reaction cycles and purified using DNA clean beads (Vazyme, N411). Final DNA libraries were quantified with a Qubit fluorometer (Invitrogen, Q32851) and sequenced using NovaS4‐150PE. Raw sequencing reads were processed using fastp for cleaning^[^ ^76^ ^]^ and mapped to the kiwifruit genome using bowtie2.^[^ ^106^ ^]^ The resulting BAM files were deduplicated with sambamba^[^ ^107^ ^]^ and converted to.bw files using bamCoverage with the parameters –normalizeUsing RPKM and –effectiveGenomeSize 608327852.^[^ ^108^ ^]^

### Statistics and Data Visualization

If not specified, R (https://cran.r‐project.org/) was used to compute statistics and generate plots. Spearman correlation was used in Figure 1A. The hypergeometric test was used in Figure 2C. The wilcoxon test was used in Figures 4D and S8F, Supporting Information. F‐test was used in Figure 4B and Figure 5D. The *t*‐test with Bonferroni correction was used in Figure 5E, Figures S12A and S18, Supporting Information.

## Conflict of Interest

The authors declare no conflict of interest.

## Author Contributions

N.M., X.L., and D.C contributed equally to this work. H.H., D.L., and N.M. conceived and designed the project. N.M. performed the experiments and bioinformatics analyses. X.L. assisted with Hi‐C data retrieving and analysis. D.C. assisted with immunofluorescence and processed confocal microscopy images. H.Z. conducted phylogenetic analyses of *patatin* genes. D.L., C.Z., and X.X. provided materials. N.M. drafted the manuscript. H.H. and X.W.D. revised the manuscript and supervised the project.

## Supporting information



Supporting Information

## Data Availability

WGBS, CUT&Tag, ATAC, and RNA‐seq sequencing reads were submitted to Sequence Read Archive (SRA) under accession number PRJNA1039995. Hi‐C raw data have been deposited in Genome Sequence Archive (GSA) under accession number CRA013743 (https://ngdc.cncb.ac.cn/gsa/browse/CRA013743). Raw data are listed in Supplemental Datasets. Code are available upon request.

## References

[1] C. R. Bauer , T. A. Hartl , G. Bosco , PLoS Genet. 2012, 8, e1002873. 22956908 10.1371/journal.pgen.1002873 PMC3431300

[2] T. Sakamoto , Y. Sakamoto , S. Grob , D. Slane , T. Yamashita , N. Ito , Y. Oko , T. Sugiyama , T. Higaki , S. Hasezawa , M. Tanaka , A. Matsui , M. Seki , T. Suzuki , U. Grossniklaus , S. Matsunaga , Nat. Plants 2022, 8, 940. 35915144 10.1038/s41477-022-01200-3

[3] C. Municio , W. Antosz , K. D. Grasser , E. Kornobis , M. Van Bel , I. Eguinoa , F. Coppens , A. Bräutigam , I. Lermontova , A. Bruckmann , K. Zelkowska , A. Houben , V. Schubert , New Phytol. 2021, 230, 972. 33475158 10.1111/nph.17221

[4] T. A. Hartl , H. F. Smith , G. Bosco , Science 2008, 322, 1384. 19039137 10.1126/science.1164216

[5] C. Hoencamp , O. Dudchenko , A. M. O. Elbatsh , S. Brahmachari , J. A. Raaijmakers , T. van Schaik , Á. Sedeño Cacciatore , V. G. Contessoto , R. G. H. P. van Heesbeen , B. van den Broek , A. N. Mhaskar , H. Teunissen , B. G. St Hilaire , D. Weisz , A. D. Omer , M. Pham , Z. Colaric , Z. Yang , S. S. P. Rao , N. Mitra , C. Lui , W. Yao , R. Khan , L. L. Moroz , A. Kohn , J. St Leger , A. Mena , K. Holcroft , M. C. Gambetta , F. Lim , et al., Science 2021, 372, 984. 34045355 10.1126/science.abe2218 PMC8172041

[6] E. Lieberman‐Aiden , N. L. van Berkum , L. Williams , M. Imakaev , T. Ragoczy , A. Telling , I. Amit , B. R. Lajoie , P. J. Sabo , M. O. Dorschner , R. Sandstrom , B. Bernstein , M. A. Bender , M. Groudine , A. Gnirke , J. Stamatoyannopoulos , L. A. Mirny , E. S. Lander , J. Dekker , Science 2009, 326, 289. 19815776 10.1126/science.1181369 PMC2858594

[7] E. M. Hildebrand , J. Dekker , Trends Biochem. Sci. 2020, 45, 385. 32311333 10.1016/j.tibs.2020.01.002 PMC7275117

[8] L. Wang , Y. Gao , X. Zheng , C. Liu , S. Dong , Ru Li , G. Zhang , Y. Wei , H. Qu , Y. Li , C. D Allis , G. Li , H. Li , P. Li , Mol. Cell 2019, 76, 646. 31543422 10.1016/j.molcel.2019.08.019

[9] S. Zhao , L. Cheng , Y. Gao , B. Zhang , X. Zheng , L. Wang , P. Li , Q. Sun , H. Li , Cell Res. 2019, 29, 54. 30425322 10.1038/s41422-018-0104-9 PMC6318295

[10] G. Fudenberg , M. Imakaev , C. Lu , A. Goloborodko , N. Abdennur , L. A. Mirny , Cell Rep. 2016, 15, 2038. 27210764 10.1016/j.celrep.2016.04.085 PMC4889513

[11] S. S. P. Rao , M. H. Huntley , N. C. Durand , E. K. Stamenova , I. D. Bochkov , J. T. Robinson , A. L. Sanborn , I. Machol , A. D. Omer , E. S. Lander , E. L. Aiden , Cell 2014, 159, 1665. 25497547 10.1016/j.cell.2014.11.021 PMC5635824

[12] Z. Shi , H. Gao , X. C. Bai , H. Yu , Science 2020, 368, 1454. 32409525 10.1126/science.abb0981

[13] J. M. Peters , A. Tedeschi , J. Schmitz , Genes Dev. 2008, 22, 3089. 19056890 10.1101/gad.1724308

[14] Y. Li , J. H. I. Haarhuis , Á. Sedeño Cacciatore , R. Oldenkamp , M. S. van Ruiten , L. Willems , H. Teunissen , K. W. Muir , E. de Wit , B. D. Rowland , D. Panne , Nature 2020, 578, 472. 31905366 10.1038/s41586-019-1910-z PMC7035113

[15] J. Nuebler , G. Fudenberg , M. Imakaev , N. Abdennur , L. A. Mirny , Proc. Natl. Acad. Sci. U. S. A. 2018, 115, E6697. 29967174 10.1073/pnas.1717730115 PMC6055145

[16] J. Weischenfeldt , T. Dubash , A. P. Drainas , B. R. Mardin , Y. Chen , A. M. Stütz , S. M. Waszak , G. Bosco , A. R. Halvorsen , B. Raeder , T. Efthymiopoulos , S. Erkek , C. Siegl , H. Brenner , O. T. Brustugun , S. M. Dieter , P. A. Northcott , I. Petersen , S. M. Pfister , M. Schneider , S. K. Solberg , E. Thunissen , W. Weichert , T. Zichner , R. Thomas , M. Peifer , A. Helland , C. R. Ball , M. Jechlinger , R. Sotillo , et al., Nat. Genet. 2017, 49, 65. 27869826 10.1038/ng.3722 PMC5791882

[17] D. Hnisz , A. S. Weintraub , D. S. Day , A. L. Valton , R. O. Bak , C. H. Li , J. Goldmann , B. R. Lajoie , Z. i. P Fan , A. A. Sigova , J. Reddy , D. Borges‐Rivera , T. I. Lee , R. Jaenisch , M. H. Porteus , J. Dekker , R. A. Young , Science 2016, 351, 1454. 26940867 10.1126/science.aad9024 PMC4884612

[18] M. Peifer , F. Hertwig , F. Roels , D. Dreidax , M. Gartlgruber , R. Menon , A. Krämer , J. L. Roncaioli , F. Sand , J. M. Heuckmann , F. Ikram , R. Schmidt , S. Ackermann , A. Engesser , Y. Kahlert , W. Vogel , J. Altmüller , P. Nürnberg , J. Thierry‐Mieg , D. Thierry‐Mieg , A. Mariappan , S. Heynck , E. Mariotti , K. O. Henrich , C. Gloeckner , G. Bosco , I. Leuschner , M. R. Schweiger , L. Savelyeva , S. C. Watkins , et al., Nature 2015, 526, 700. 26466568 10.1038/nature14980 PMC4881306

[19] F. Dong , J. Jiang , Chromosome Res. 1998, 6, 551. 9886774 10.1023/a:1009280425125

[20] P. Dong , X. Tu , Po‐Yu Chu , P. Lü , N. Zhu , D. Grierson , B. Du , P. Li , S. Zhong , Mol. Plant 2017, 10, 1497. 29175436 10.1016/j.molp.2017.11.005

[21] E. S. Dogan , C. Liu , Nat Plants 2018, 4, 521. 30061747 10.1038/s41477-018-0199-5

[22] M. Srinivasan , J. C. Scheinost , N. J. Petela , T. G. Gligoris , M. Wissler , S. Ogushi , J. E. Collier , M. Voulgaris , A. Kurze , K. L. Chan , B. Hu , V. Costanzo , K. A. Nasmyth , Cell 2018, 173, 1508. 29754816 10.1016/j.cell.2018.04.015 PMC6371919

[23] K. A. Hagstrom , B. J. Meyer , Nat. Rev. Genet. 2003, 4, 520. 12838344 10.1038/nrg1110

[24] Yu Zhang , M. Ma , M. Liu , A. Sun , X. Zheng , K. Liu , C. Yin , C. Li , C. Jiang , X. Tu , Y. Fang , Nat. Commun. 2023, 14, 1209. 36869051 10.1038/s41467-023-36788-3 PMC9984397

[25] L. Jiang , M. Xia , L. I. Strittmatter , C. A. Makaroff , Plant J. 2007, 50, 1020. 17488242 10.1111/j.1365-313X.2007.03106.x

[26] E. S. Karaaslan , N. Wang , N. Faiß , Y. Liang , S. A. Montgomery , S. Laubinger , K. W. Berendzen , F. Berger , H. Breuninger , C. Liu , Nat. Plants 2020, 6, 1250. 32895530 10.1038/s41477-020-00766-0

[27] X. Li , X. Tang , X. Bing , C. Catalano , T. Li , G. Dolsten , C. Wu , M. Levine , Mol. Cell 2023, 83, 1519. 37003261 10.1016/j.molcel.2023.03.011 PMC10396332

[28] M. Louwers , R. Bader , M. Haring , R. van Driel , W. de Laat , M. Stam , Plant Cell 2009, 21, 832. 19336692 10.1105/tpc.108.064329 PMC2671708

[29] P. Crevillen , C. Sonmez , Z. Wu , C. Dean , EMBO J. 2013, 32, 140. 23222483 10.1038/emboj.2012.324 PMC3545306

[30] S. Li , D. Lin , Y. Zhang , M. Deng , Y. Chen , B. Lv , B. Li , Y. Lei , Y. Wang , L. Zhao , Y. Liang , J. Liu , K. Chen , Z. Liu , J. Xiao , J. L. Qiu , C. Gao , Nature 2022, 602, 455. 35140403 10.1038/s41586-022-04395-9

[31] E. Wegel , R. Koumproglou , P. Shaw , A. Osbourn , Plant Cell 2009, 21, 3926. 20040536 10.1105/tpc.109.072124 PMC2814510

[32] H. W. Nützmann , D. Doerr , A. Ramírez‐Colmenero , J. E. Sotelo‐Fonseca , E. Wegel , M. Di Stefano , S. W. Wingett , P. Fraser , L. Hurst , S. L. Fernandez‐Valverde , A. Osbourn , Proc. Natl. Acad. Sci. U. S. A. 2020, 117, 13800. 32493747 10.1073/pnas.1920474117 PMC7306824

[33] V. S. Lioy , J. N. Lorenzi , S. Najah , T. Poinsignon , H. Leh , C. Saulnier , B. Aigle , S. Lautru , A. Thibessard , O. Lespinet , P. Leblond , Y. Jaszczyszyn , K. Gorrichon , N. Varoquaux , I. Junier , F. Boccard , J. L. Pernodet , S. Bury‐Moné , Nat. Commun. 2021, 12, 5221. 34471117 10.1038/s41467-021-25462-1 PMC8410849

[34] W. Zhang , L. Lazar‐Stefanita , H. Yamashita , M. J. Shen , L. A. Mitchell , H. Kurasawa , E. Lobzaev , V. Fanfani , M. A. B. Haase , X. Sun , Q. Jiang , G. W. Goldberg , D. M. Ichikawa , S. L. Lauer , L. H. McCulloch , N. Easo , S. J Lin , B. R. Camellato , Y. Zhu , J. Cai , Z. Xu , Yu Zhao , M. Sacasa , R. Accardo , L. A. Brammer Basta , N. R. Bello , L. Cai , S. Cerritos , M. Cornwell , A. D'Amato , et al., Mol. Cell 2023, 83, 4424. 37944526 10.1016/j.molcel.2023.10.015

[35] L‐Ge Chen , T. Lan , S. Zhang , M. Zhao , G. Luo , Yi Gao , Y. Zhang , Q. Du , H. Lu , B. Li , B. Jiao , Z. Hu , Y. Ma , Q. Zhao , Y. Wang , W. Qian , J. Dai , Y. Jiao , Nat. Plants 2024, 10, 228. 38278952 10.1038/s41477-023-01595-7

[36] X. Han , Y. Zhang , Q. Zhang , Ni Ma , X. Liu , W. Tao , Z. Lou , C. Zhong , X. W. Deng , D. Li , H. He , Mol. Plant 2023, 16, 452. 36588343 10.1016/j.molp.2022.12.022

[37] J. Jia , Y. Xie , J. Cheng , C. Kong , M. Wang , L. Gao , F. Zhao , J. Guo , K. Wang , G. Li , D. Cui , T. Hu , G. Zhao , D. Wang , Z. Ru , Y. Zhang , Genome Biol. 2021, 22, 1. 33419466 10.1186/s13059-020-02225-7 PMC7792079

[38] Yi Liao , J. Wang , Z. Zhu , Y. Liu , J. Chen , Y. Zhou , F. Liu , J. Lei , B. S. Gaut , B. Cao , J. J. Emerson , C. Chen , Nat. Commun. 2022, 13, 3479. 35710823 10.1038/s41467-022-31112-x PMC9203530

[39] L. Concia , A. Veluchamy , J. S. Ramirez‐Prado , A. Martin‐Ramirez , Y. Huang , M. Perez , S. Domenichini , N. Y. Rodriguez Granados , S. Kim , T. Blein , S. Duncan , C. Pichot , D. Manza‐Mianza , C. Juery , E. Paux , G. Moore , H. Hirt , C. Bergounioux , M. Crespi , M. M. Mahfouz , A. Bendahmane , C. Liu , A. Hall , C. Raynaud , D. Latrasse , M. Benhamed , Genome Biol. 2020, 21, 104. 32349780 10.1186/s13059-020-01998-1 PMC7189446

[40] P. Fransz , J. H. De Jong , M. Lysak , M. R. Castiglione , I. Schubert , Proc. Natl. Acad. Sci. U. S. A. 2002, 99, 14584. 12384572 10.1073/pnas.212325299 PMC137926

[41] X. Yang , L. Zhang , X. Guo , J. Xu , K. Zhang , Y. Yang , Yu Yang , Y. Jian , D. Dong , S. Huang , F. Cheng , G. Li , Mol. Plant 2023, 16, 314. 36528795 10.1016/j.molp.2022.12.010

[42] R. C. O'Malley , S. S. C. Huang , L. Song , M. G. Lewsey , A. Bartlett , J. R. Nery , M. Galli , A. Gallavotti , J. R. Ecker , Cell 2016, 165, 1280. 27203113 10.1016/j.cell.2016.04.038 PMC4907330

[43] M. Kooiker , C. A. Airoldi , A. Losa , P. S. Manzotti , L. Finzi , M. M. Kater , L. Colombo , Plant Cell 2005, 17, 722. 15722463 10.1105/tpc.104.030130 PMC1069694

[44] S. Simonini , I. Roig‐Villanova , V. Gregis , B. Colombo , L. Colombo , M. M. Kater , Plant Cell 2012, 24, 4163. 23054472 10.1105/tpc.112.103952 PMC3517243

[45] K. Domb , N. Wang , G. Hummel , C. Liu , Annu. Rev. Plant Biol. 2022, 73, 173. 35130445 10.1146/annurev-arplant-102720-022810

[46] X. Lan , J. K. Pritchard , Science 2016, 352, 1009. 27199432 10.1126/science.aad8411 PMC5182070

[47] X. M. Lu , X. F. Yu , G. Q. Li , M. H. Qu , H. Wang , C. Liu , et al. Plant Commun. 2024, 5, 100856. 38431772 10.1016/j.xplc.2024.100856 PMC11211551

[48] X. Yao , Mol. Hortic. 2022, 2, 13. 37789488 10.1186/s43897-022-00034-z PMC10515239

[49] Y. Jiao , N. J. Wickett , S. Ayyampalayam , A. S. Chanderbali , L. Landherr , P. E. Ralph , L. P. Tomsho , Yi Hu , H. Liang , P. S. Soltis , D. E. Soltis , S. W. Clifton , S. E. Schlarbaum , S. C. Schuster , H. Ma , J. Leebens‐Mack , C. W. dePamphilis , Nature 2011, 473, 97. 21478875 10.1038/nature09916

[50] Y. Wang , H. Tang , J. D. DeBarry , X. Tan , J. Li , X. Wang , T.‐H. Lee , H. Jin , B. Marler , H. Guo , J. C. Kissinger , A. H. Paterson , Nucleic Acids Res. 2012, 40, e49. 22217600 10.1093/nar/gkr1293 PMC3326336

[51] S. Maere , S. De Bodt , J. Raes , T. Casneuf , M. Van Montagu , M. Kuiper , Y. Van de Peer , Proc. Natl. Acad. Sci. U. S. A. 2005, 102, 5454. 15800040 10.1073/pnas.0501102102 PMC556253

[52] M. Lynch , J. S. Conery , Science 2000, 290, 1151. 11073452 10.1126/science.290.5494.1151

[53] D. Pan , L. Zhang , Genome Biol. 2007, 8, R158. 17683522 10.1186/gb-2007-8-8-r158 PMC2374989

[54] T. Shi , H. Huang , M. S. Barker , Ann. Bot. 2010, 106, 497. 20576738 10.1093/aob/mcq129 PMC2924827

[55] K. M. Teshima , H. Innan , Genetics 2004, 166, 1553. 15082568 10.1534/genetics.166.3.1553 PMC1470786

[56] Fu‐S Yang , S. Nie , H. Liu , T‐Le Shi , X. C. Tian , S. S. Zhou , Yu‐T Bao , K. H. Jia , J. F. Guo , W. Zhao , Na An , R. G. Zhang , Q. Z. Yun , X. Z. Wang , C. Mannapperuma , I. Porth , Y. A. El‐Kassaby , N. R. Street , X‐Ru Wang , Y. Van de Peer , J. F. Mao , Nat. Commun. 2020, 11, 5269. 33077749 10.1038/s41467-020-18771-4 PMC7572368

[57] S. Nie , S. W. Zhao , T‐Le Shi , W. Zhao , R. G. Zhang , X. C. Tian , J. F. Guo , X. M. Yan , Yu‐T Bao , Z. C. Li , L. Kong , H. Y. Ma , Z. Y. Chen , H. Liu , Y. A. El‐Kassaby , I. Porth , Fu‐S Yang , J. F. Mao , Hortic. Res. 2023, 10, uhac241. 36643737 10.1093/hr/uhac241 PMC9832866

[58] W. Nagl , H.‐P. Fusenig in Genome and Chromatin: Organization, Evolution, Function: Symposium, Springer, Kaiserslautern, 1978, 221.

[59] W. Shan , M. Kubova , T. Mandakova , M. A. Lysak , Plant J. 2021, 108, 528. 34390055 10.1111/tpj.15459

[60] H. X. Cao , G. T. Vu , W. Wang , J. Messing , I. Schubert , Plant Biol. (Stuttg) 2015, 17, 120. 24853858 10.1111/plb.12194

[61] H. Lu , D. Yu , A. S. Hansen , S. Ganguly , R. Liu , A. Heckert , X. Darzacq , Q. Zhou , Nature 2018, 558, 318. 29849146 10.1038/s41586-018-0174-3 PMC6475116

[62] W‐Ki Cho , J. H. Spille , M. Hecht , C. Lee , C. Li , V. Grube , I. I. Cisse , Science 2018, 361, 412. 29930094 10.1126/science.aar4199 PMC6543815

[63] A. Bancaud , S. Huet , N. Daigle , J. Mozziconacci , J. Beaudouin , J. Ellenberg , EMBO J. 2009, 28, 3785. 19927119 10.1038/emboj.2009.340 PMC2797059

[64] K. Richter , M. Nessling , P. Lichter , J. Cell Sci. 2007, 120, 1673. 17430977 10.1242/jcs.03440

[65] M. Falk , Y. Feodorova , N. Naumova , M. Imakaev , B. R. Lajoie , H. Leonhardt , B. Joffe , J. Dekker , G. Fudenberg , I. Solovei , L. A. Mirny , Nature 2019, 570, 395. 31168090 10.1038/s41586-019-1275-3 PMC7206897

[66] H. Cao , J. Chen , M. Yue , C. Xu , W. Jian , Y. Liu , B. Song , Y. Gao , Y. Cheng , Z. Li , Plant J. 2020, 104, 1568. 33048422 10.1111/tpj.15021

[67] C. J. Doherty , H. A. Van Buskirk , S. J. Myers , M. F. Thomashow , Plant Cell 2009, 21, 972. 19270186 10.1105/tpc.108.063958 PMC2671710

[68] W. H. Tian , J. Y. Ye , M. Qi Cui , J. Bo Chang , Yu Liu , G. X. Li , Y. R. Wu , Ji. M Xu , N. P. Harberd , C. Z. Mao , C. W. Jin , Z. J. Ding , S. J. Zheng , Mol. Plant 2021, 14, 1554. 34216828 10.1016/j.molp.2021.06.024

[69] D. Leister , Trends Genet. 2004, 20, 116. 15049302 10.1016/j.tig.2004.01.007

[70] K. Choi , X. Zhao , K. A. Kelly , O. Venn , J. D. Higgins , N. E. Yelina , T. J. Hardcastle , P. A. Ziolkowski , G. P. Copenhaver , F. C. H. Franklin , G. McVean , I. R. Henderson , Nat. Genet. 2013, 45, 1327. 24056716 10.1038/ng.2766 PMC3812125

[71] X. Jin , G. Fudenberg , K. S. Pollard , Genome Res. 2021, 31, 1561. 34301629 10.1101/gr.275358.121 PMC8415379

[72] A. A. Golicz , P. L. Bhalla , D. Edwards , M. B. Singh , Commun. Biol. 2020, 3, 235. 32398676 10.1038/s42003-020-0932-2 PMC7217851

[73] K. Bomblies , J. D. Higgins , L. Yant , New Phytol. 2015, 208, 306. 26075313 10.1111/nph.13499

[74] N. Naumova , M. Imakaev , G. Fudenberg , Ye Zhan , B. R. Lajoie , L. A. Mirny , J. Dekker , Science 2013, 342, 948. 24200812 10.1126/science.1236083 PMC4040465

[75] Y. Wang , H. Wang , Yu Zhang , Z. Du , W. Si , S. Fan , D. Qin , M. Wang , Y. Duan , L. Li , Y. Jiao , Y. Li , Q. Wang , Q. Shi , X. Wu , W. Xie , Mol. Cell 2019, 73, 547. 30735655 10.1016/j.molcel.2018.11.019

[76] S. Chen , Y. Zhou , Y. Chen , J. Gu , Bioinformatics 2018, 34, i884. 30423086 10.1093/bioinformatics/bty560 PMC6129281

[77] N. Servant , N. Varoquaux , B. R. Lajoie , E. Viara , C. J. Chen , J. P. Vert , E. Heard , J. Dekker , E. Barillot , Genome Biol. 2015, 16, 1. 26619908 10.1186/s13059-015-0831-x PMC4665391

[78] D. Lin , J. Sanders , W. S. Noble , HiCRep. Bioinformatics 2021, 37, 2996. 33576390 10.1093/bioinformatics/btab097 PMC8479650

[79] N. C. Durand , J. T. Robinson , M. S. Shamim , I. Machol , J. P. Mesirov , E. S. Lander , E. L. Aiden , Cell Syst. 2016, 3, 99. 27467250 10.1016/j.cels.2015.07.012 PMC5596920

[80] J. Wolff , V. Bhardwaj , S. Nothjunge , G. Richard , G. Renschler , R. Gilsbach , T. Manke , R. Backofen , F. Ramírez , B. A. Grüning , Nucleic Acids Res. 2018, 46, W11. 29901812 10.1093/nar/gky504 PMC6031062

[81] M. Naish , M. Alonge , P. Wlodzimierz , A. J. Tock , B. W. Abramson , A. Schmücker , T. Mandáková , B. Jamge , C. Lambing , P. Kuo , N. Yelina , N. Hartwick , K. Colt , L. M. Smith , J. Ton , T. Kakutani , R. A. Martienssen , K. Schneeberger , M. A. Lysak , F. Berger , A. Bousios , T. P. Michael , M. C. Schatz , I. R. Henderson , Science 2021, 374, eabi7489. 34762468 10.1126/science.abi7489 PMC10164409

[82] L. Shang , W. He , T. Wang , Y. Yang , Q. Xu , X. Zhao , L. Yang , H. Zhang , X. Li , Y. Lv , Wu Chen , S. Cao , X. Wang , B. Zhang , X. Liu , X. Yu , H. He , H. Wei , Y. Leng , C. Shi , M. Guo , Z. Zhang , B. Zhang , Q. Yuan , H. Qian , X. Cao , Y. Cui , Q. Zhang , X. Dai , C. Liu , et al., Mol. Plant 2023, 16, 1232. 37553831 10.1016/j.molp.2023.08.003

[83] Y. Zhou , Z. Zhang , Z. Bao , H. Li , Y. Lyu , Y. Zan , Y. Wu , L. Cheng , Y. Fang , K. Wu , J. Zhang , H. Lyu , T. Lin , Q. Gao , S. Saha , L. Mueller , Z. Fei , T. Städler , S. Xu , Z. Zhang , D. Speed , S. Huang , Nature 2022, 606, 527. 35676474 10.1038/s41586-022-04808-9 PMC9200638

[84] L. Wang , M. Zhang , M. Li , X. Jiang , Wu Jiao , Q. Song , Mol. Plant 2023, 16, 1711. 37634078 10.1016/j.molp.2023.08.012

[85] A. Song , J. Su , H. Wang , Z. Zhang , X. Zhang , Y. Van de Peer , F. Chen , W. Fang , Z. Guan , F. Zhang , Z. Wang , L. Wang , B. Ding , S. Zhao , L. Ding , Ye Liu , L. Zhou , J. He , D. Jia , J. Zhang , C. Chen , Z. Yu , D. Sun , J. Jiang , S. Chen , F. Chen , Nat. Commun. 2023, 14, 2021. 37037808 10.1038/s41467-023-37730-3 PMC10085997

[86] P. X. Xiao , Y. Li , J. Lu , H. Zuo , G. Pingcuo , H. Ying , F. Zhao , Q. Xu , X. Zeng , W. B. Jiao , Hortic. Res. 2023, 10, uhad241. 38156287 10.1093/hr/uhad241 PMC10753165

[87] F. Hao , X. Liu , B. Zhou , Z. Tian , L. Zhou , H. Zhong , J. Qi , J. He , Y. Zhang , P. Zeng , Q. Li , K. Wang , K. Xia , X. Gou , L. Li , W. Shao , B. Zhang , S. Li , H. Yang , L. Hui , W. Chen , L. Peng , F. Liu , Z.‐Q. Rong , Y. Peng , W. Zhu , J. A. McCallum , Z. Li , X. Xu , H. Yang , et al., Nat. Genet. 2023, 55, 1976. 37932434 10.1038/s41588-023-01546-0

[88] X. Shi , S. Cao , Xu Wang , S. Huang , Y. Wang , Z. Liu , W. Liu , X. Leng , Y. Peng , N. Wang , Y. Wang , Z. Ma , X. Xu , F. Zhang , H. Xue , H. Zhong , Yi Wang , K. Zhang , A. Velt , K. Avia , D. Holtgräwe , J. Grimplet , J. T. Matus , D. Ware , X. Wu , H. Wang , C. Liu , Y. Fang , C. Rustenholz , Z. Cheng , et al., Hortic. Res. 2023, 10, uhad061. 37213686 10.1093/hr/uhad061 PMC10199708

[89] F. Ramírez , V. Bhardwaj , L. Arrigoni , K. C. Lam , B. A. Grüning , J. Villaveces , B. Habermann , A. Akhtar , T. Manke , Nat. Commun. 2018, 9, 189. 29335486 10.1038/s41467-017-02525-w PMC5768762

[90] O. Dudchenko , S. S. Batra , A. D. Omer , S. K. Nyquist , M. Hoeger , N. C. Durand , M. S. Shamim , I. Machol , E. S. Lander , A. P. Aiden , E. L. Aiden , Science 2017, 356, 92. 28336562 10.1126/science.aal3327 PMC5635820

[91] P. Langfelder , S. Horvath , BMC Bioinformatics 2008, 9, 559. 19114008 10.1186/1471-2105-9-559 PMC2631488

[92] L. Lopez‐Delisle , L. Rabbani , J. Wolff , V. Bhardwaj , R. Backofen , B. Grüning , F. Ramírez , T. Manke , Bioinformatics 2021, 37, 422. 32745185 10.1093/bioinformatics/btaa692 PMC8058774

[93] J. C. Stansfield , K. G. Cresswell , M. G. Dozmorov , Bioinformatics 2019, 35, 2916. 30668639 10.1093/bioinformatics/btz048 PMC6736119

[94] Z. Gu , L. Gu , R. Eils , M. Schlesner , B. Brors , Bioinformatics 2014, 30, 2811. 24930139 10.1093/bioinformatics/btu393

[95] I. M. Flyamer , R. S. Illingworth , W. A. Bickmore , Bioinformatics 2020, 36, 2980. 32003791 10.1093/bioinformatics/btaa073 PMC7214034

[96] C. E. Grant , T. L. Bailey , W. S. Noble , Bioinformatics 2011, 27, 1017. 21330290 10.1093/bioinformatics/btr064 PMC3065696

[97] Z. Zhang , J. Xiao , J. Wu , H. Zhang , G. Liu , X. Wang , L. Dai , Biochem. Biophys. Res. Commun. 2012, 419, 779. 22390928 10.1016/j.bbrc.2012.02.101

[98] W. He , J. Yang , Y. Jing , L. Xu , K. Yu , X. Fang , Bioinformatics 2023, 39, btad121. 36883694 10.1093/bioinformatics/btad121 PMC10027429

[99] D. M. Emms , S. Kelly , Genome Biol. 2019, 20, 238. 31727128 10.1186/s13059-019-1832-y PMC6857279

[100] R. C. Edgar , Nucleic Acids Res. 2004, 32, 1792. 15034147 10.1093/nar/gkh340 PMC390337

[101] A. Stamatakis , Bioinformatics 2014, 30, 1312. 24451623 10.1093/bioinformatics/btu033 PMC3998144

[102] K. Tamura , G. Stecher , S. Kumar , Mol. Biol. Evol. 2021, 38, 3022. 33892491 10.1093/molbev/msab120 PMC8233496

[103] Z. Yang , Mol. Biol. Evol. 2007, 24, 1586. 17483113 10.1093/molbev/msm088

[104] S. Kumar , G. Stecher , M. Suleski , S. B. T. T. A. R. T Hedges , Mol. Biol. Evol. 2017, 34, 1812. 28387841 10.1093/molbev/msx116

[105] Y. Zhao , H. Deng , Y. Chen , J. Li , S. Chen , C. Li , X. Mu , Z. Hu , K. Li , W. Wang , Front. Plant Sci. 2022, 13, 906168. 35734244 10.3389/fpls.2022.906168 PMC9208197

[106] B. Langmead , S. L. Salzberg , Nat. Methods 2012, 9, 357. 22388286 10.1038/nmeth.1923 PMC3322381

[107] A. Tarasov , A. J. Vilella , E. Cuppen , I. J. Nijman , P. Prins , Bioinformatics 2015, 31, 2032. 25697820 10.1093/bioinformatics/btv098 PMC4765878

[108] F. Ramírez , D. P. Ryan , B. Grüning , V. Bhardwaj , F. Kilpert , A. S. Richter , S. Heyne , F. Dündar , T. Manke , Nucleic Acids Res. 2016, 44, W160. 27079975 10.1093/nar/gkw257 PMC4987876

