# Supplementary material for: Chromatin Topological Domains Associate With the Rapid Formation of Tandem Duplicates in Plants
Source: Adv Sci (Weinh). 2024 Dec 27;12(7):2408861. doi: 10.1002/advs.202408861 (PMC11831494; doi:10.1002/advs.202408861)
Supplement: Supplementary file 1 — Supporting Information [file ADVS-12-2408861-s001.docx]

Supporting Information

**Chromatin topological domain accelerates the origination of tandem duplicates in plants**

*Ni Ma^1,2,4^, Xiaopeng Li^2,4^, Dong Ci^1,2,4^, Haiyue Zeng^1,2^, Congxiao Zhang^3^, Xiaodong Xie^3^, Caihong Zhong^3^, Xing Wang Deng^1,2*^, Dawei Li^3*^, and Hang He^1,2*^*

^4^ These authors contributed equally to this work.

Correspondence: Xing Wang Deng (deng@pku.edu.cn), Dawei Li (lidawei@wbgcas.cn), Hang He (hehang@pku.edu.cn)

**Handling giant chromosome during Hi-C data analysis**

For tree peony, chromosome 1,2,4 were each split into two small chromosomes (eg. Chr1.1 and Chr1.2). The resulting assembly file was used for reads mapping during hic-pro pipeline. For visualization, the .validPairs was converted to .hic file. For PC1 calculation, genomic blocks recorded in .abs.bed file were re-assembled, and scaled to 1/10 of original chromosome length. Precisely speaking, the coordinates for split chromosomes (Chr1.2, Chr2.2, Chr4.2) were renumbered to follow their progenitors (Chr1.1, Chr2.1, Chr4.1). In this way we obtained the bed file recording the original whole chromosome. Next, in our 500 kb-resolution hic-pro matrix, the .abs.bed file recorded 500 kb-long interval windows across the genome. To limit the positional coordinates to 2^31 bp, the 500 kb-long intervals were adjusted to 50 kb-long ones. Namely, the ~2 Gb giant chromosomes were scaled to ~ 200 Mb. Then the .abs.bed file together with the matrix file was converted to .cool file, and PC1 values were computed by hicPCA.

**ATAC and data processing**

We extracted kiwifruit nuclei as described before. ~ 50,000 nuclei were incubated with Tn5 mix (Novoprotein, N248) at 37 °C for 30 min. The library was purified with DNA clean beads (Novoprotein, N240) and sequenced with NovaS4-150PE. Sequencing reads were trimmed and mapped, and low-quality alignments were filtered by ‘samtools view -q 20’. After deduplication, the bam file was converted to .bw file. The .bw file was visualized at certain genome regions by deeptools^1^ utility ‘computeMatrix’ and ‘plotHeatmap’. ATAC peaks were called by macs3^2^ with parameter setting ‘--nomodel -q 0.01 --shift -50 --extsize 100’.

**WGBS data processing**

The existing genome assembly of *A. chinensis* var ‘Donghong’ was complemented with kiwifruit chloroplast DNA^3^. Whole-genome bisulfite sequencing reads were mapped to the complete genome following methylpy^4^ pipeline. The generated .allc files were converted to .bed files by ‘allc-to-bigwig’, and the spearman correlation coefficients between each pair of replicates were calculated by R function ‘cor’. Having determined the experiment quality and data reproductivity, .allc files of replicates were merged, and converted to .bw files encompassing the methylation type of CG/CHG/CHH, respectively. The parameter ‘--bin-size’ was adjusted to match the Hi-C matrix resolution.

**RNA-seq data processing**

RNA-seq libraries were constructed and sequenced using the DNBSEQ-T7 platform by BGI Co., Ltd. Paired-end reads were mapped to kiwifruit genome by hisat2^5^. The alignments on each transcript were counted by FeatureCounts^6^, and were analyzed by DESeq2^7^. DEGs (differentially expressing genes) were extracted with threshold ‘log2(fold change) > 2 & p.adj < 0.01’. FPKM was calculated by Stringtie^8^.

**Differential H3K27me3 deposition analysis between tissues**

The peak files of each tissue sample were clustered by ‘bedtools cluster’ and then merged into one .gtf file. The raw CUT&Tag reads were mapped to this gtf by bowtie2^9^, and read coverage on each peak locus was obtained by FeatureCounts^6^. The peak locus with differential H3K27me3 deposition between kiwifruit leaf and root tissues were evaluated by DESeq2^7^ with parameter ‘pvalue < 0.01 & abs(res$log2FoldChange) >= 2’. Validation of the identified differential H3K27me3 peak was carried out by deeptools^1^ utility ‘computeMatrix’ and ‘plotHeatmap’.

**Chromatin domain boundary identification**

Insulation scores was calculated by cooltools^10^ under 2 kb resolution, and the window size was set from 8000 to 36000. The resulting bigwig files accompanied with local KR-normalized contact matrix were plotted by pyGenomeTrack^11^, showing that insulation scores called by 26000-window size was optimal to delineate chromatin contact pattern. With parameter setting ‘cooltools insulation --threshold Li 26000 --ignore-diags 0’, we extracted bins with relatively lowest insulation scores. These bins were defined as domain boundaries.

**Chromatin domain length calculation**

The length of the interval between two adjacent domain boundaries was defined as the length of a domain. The distribution of domain length was plotted by ‘geom_density’. To map the correlation between domain length and intergenic length, we selected intergenic regions longer than 4 kb. The length of each domain was mapped to the length of its belonging intergenic space. The point density map was plotted by ‘geom_pointdensity’.

**Feature enrichment analysis on domains and boundaries**

Euchromatin domains were called by Arrowhead under 5 kb resolution. Domains called in at least 2 tissues were kept. All genomic and epigenetic features were profiled at 2 kb resolution. The values of bins within domains/boundaries were compared with that of bins in random positions. The log2 fold change was designated as enrichment score and examined by ‘wilcox.test’ in R.

**Effect of TAD-like domain on gene co-expression**

To estimate the co-expression level of a tandem duplicate gene cluster (TDGC), we calculated the pearson correlation of FPKM across different tissue samples for each combination of gene pairs within TDGC. Namely, a TDGC with n gene copies should produce [n(n-1)]/2 data points, representing its overall co-expression level. However, as the distribution of genomic distance (*d*) and synonymous divergence (*Ks*) for the two groups of genes (within TAD and without TAD) are quite different, so we can’t say the difference in co-expression level is attributable to TAD-like domain structure. Therefrom, we have to control for *d* and *Ks*.

According to previous study, controlling for *Ks* could be achieved by segmentation^12^. However, if we add an extra layer of segmentation on *d*, the data points would be too little to support further analysis. We thus focus on **tandem** duplicates that are neighboring to each other, given that larger TDGCs within TAD-like domain have much more gene pairs with extreme separation (eg. genes on two ends of a gene cluster). In this way, 873 tandem duplicates out of 3,089 combinations of gene pairs are kept, and the deviation on distribution of *d* is considerably controlled. Finally, the effect of TAD-like domain on gene ex-regulation could be accurately probed.

|  | Hi-C data | Genome assembly |
| --- | --- | --- |
| Rice | https://doi.org/10.1016/j.molp.2017.11.005 | NipT2T |
| Tomato | https://doi.org/10.1016/j.molp.2017.11.005 | SL5.0 |
| Soybean | https://doi.org/10.1016/j.molp.2017.11.005 | GmT2T |
| Pepper | https://doi.org/10.1038/s41467-022-31112-x | CA59 |
| Arabidopsis | https://doi.org/10.1038/s41467-020-15809-5 | Col-CEN |
| Wheat | https://ngdc.cncb.ac.cn/gsa/s/xxP6N7L2 | CS-IAAS |
| Potato | https://doi.org/10.1016/j.molp.2022.12.010 | DM8.1 |
| Grape vine | https://doi.org/10.1093/hr/uhad205 | PN_T2T |
|  | https://doi.org/10.1093/hr/uhad260 |  |
| Chrysanthemum | https://doi.org/10.1038/s41467-023-37730-3 | Cmo.genome |
| Tree peony | <https://doi.org/10.1093/hr/uhad241> | Paeonia.ludlowii |
| Welsh onion | https://doi.org/10.1038/s41588-023-01546-0 | Allium.fistulosum |
| Onion |  | Allium.cepa |
| *A. arguta* | https://doi.org/10.1016/j.xplc.2024.100856 | arguta_M1 |
| *A. latifolia* | https://doi.org/10.1016/j.molp.2022.12.022 | AlKY |
| *A. chinensis* | —— | AcDH |
| *R. molle* | https://doi.org/10.1093/hr/uhac241 | RHMOLv1 |

|  | Download site for genome assembly |
| --- | --- |
| Rice | http://www.ricesuperpir.com/uploads/common/genome_sequence/NIP-T2T.fa.gz |
| Tomato | http://solomics.agis.org.cn/tomato/ftp/genome/ |
| Soybean | https://ftp.ncbi.nlm.nih.gov/genomes/all/GCA/030/864/155/GCA_030864155.1_ASM3086415v1/ |
| Pepper | https://ftp.cngb.org/pub/CNSA/data3/CNP0001129/CNS0252417/CNA0036143/ |
| Arabidopsis | https://www.arabidopsis.org/api/download-files/download?filePath=Genes/Col-CEN_genome_assembly_release/ |
| Wheat | https://zenodo.org/api/records/10716954/draft/files/CS-IAAS_v1.1_annotation.zip/ |
| Potato | http://www.bioinformaticslab.cn/pubs/dm8/DM8.1_genome.fasta.gz |
| Grape vine | https://grapedia.org/wp-content/uploads/2023/11/T2T_ref.zip |
| Chrysanthemum | https://figshare.com/ndownloader/files/39523972 |
| Tree peony | https://doi.org/10.6084/m9.figshare.23537670 |
| Welsh onion | https://download.cncb.ac.cn/gwh/Plants/Allium_fistulosum_Allium.fistulosum_GWHCBIA00000000/ |
| Onion | https://download.cncb.ac.cn/gwh/Plants/Allium_cepa_Allium_cepa_GWHCBIB00000000/ |
| *A. arguta* | https://download.cncb.ac.cn/gwh/Plants/Actinidia_arguta_M1_GWHBJWW00000000/ |
| *A. latifolia* | http://182.92.183.62/download?path=/data |
| *A. chinensis* | http://182.92.183.62/download?path=/data |
| *R. molle* | https://ftp.ncbi.nlm.nih.gov/genomes/all/GCA/025/413/875/GCA_025413875.1_RHMOLv1/ |
|  | https://github.com/JeffreyNIEgithub/RmolleGenome |

**Table S1. Table contains previously published genomics resources used**


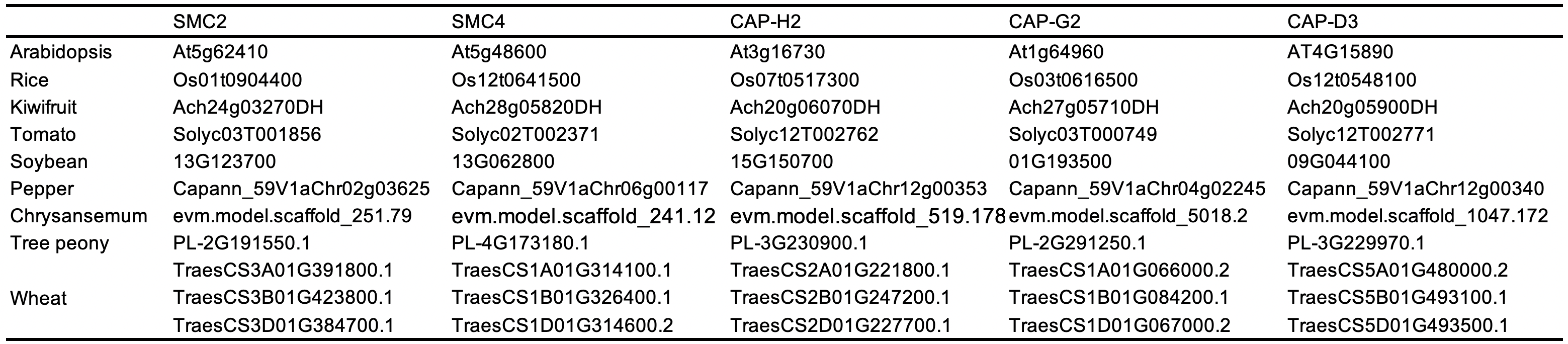


**Table S2. Conservation status of condensin II in the 9 species examined**

The peptide sequence of condensin subunits from *Arabidopsis* was used, and the best blast hit in other organisms was depicted. E-value threshold was set to 1e-20. All examined species contain a full set of condensin II subunits. Onion and welsh onion are absent in the list, for their annotation files are missing^13^.


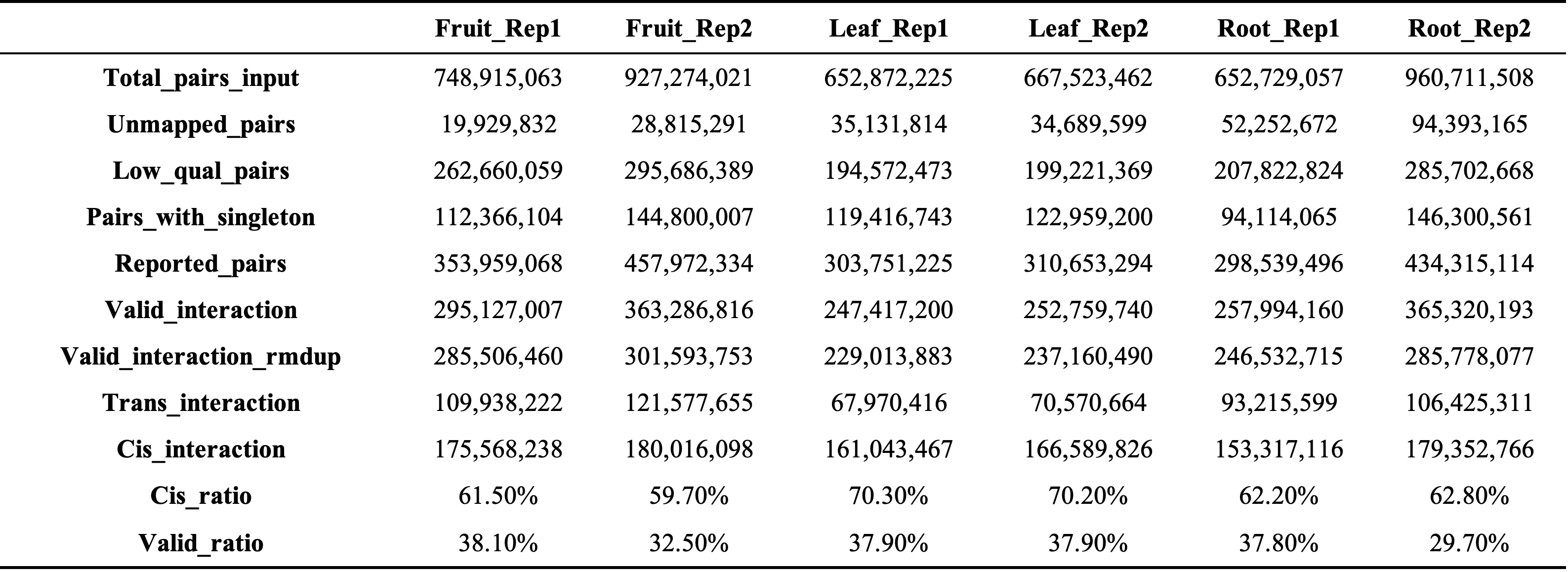


**Table S3. A summary for Hi-C data generated in this study**

Inter-chromosome contacts were defined as ‘Trans’ interaction while intra-chromosome contacts were defined as ‘Cis’ interaction. Due to the existence of chromosome territory, the Cis ratio should be greater than 50%, and a higher ratio represented a higher quality of Hi-C experiment.


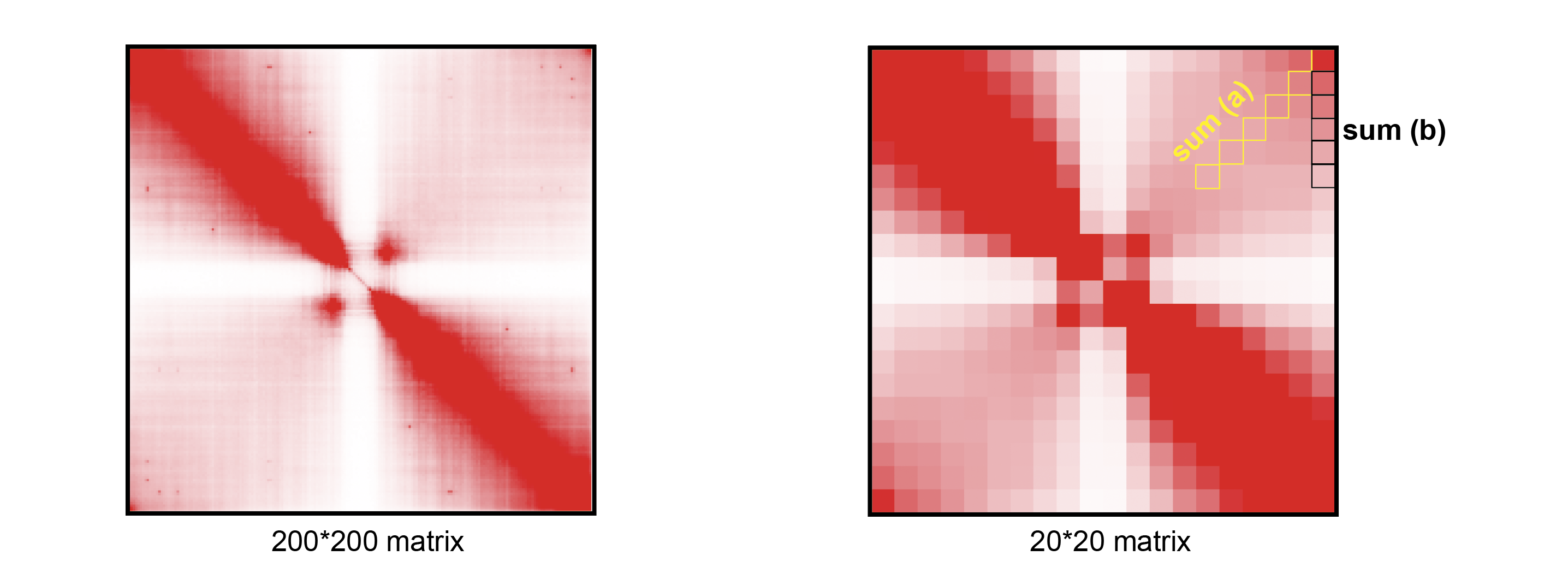


**Figure S1. Scoring for telomere-to-centromere axis in aggregated Hi-C matrix.**

The presence of enhanced contact frequency on telomere-to-centromere axis is quantified as the ‘axis score’: sum(a) / sum(b). To prevent inference from centromere compartmentalization, only the first 6 bins are counted. The ACA map used in this figure is from Arabidopsis Col-CEN assembly.


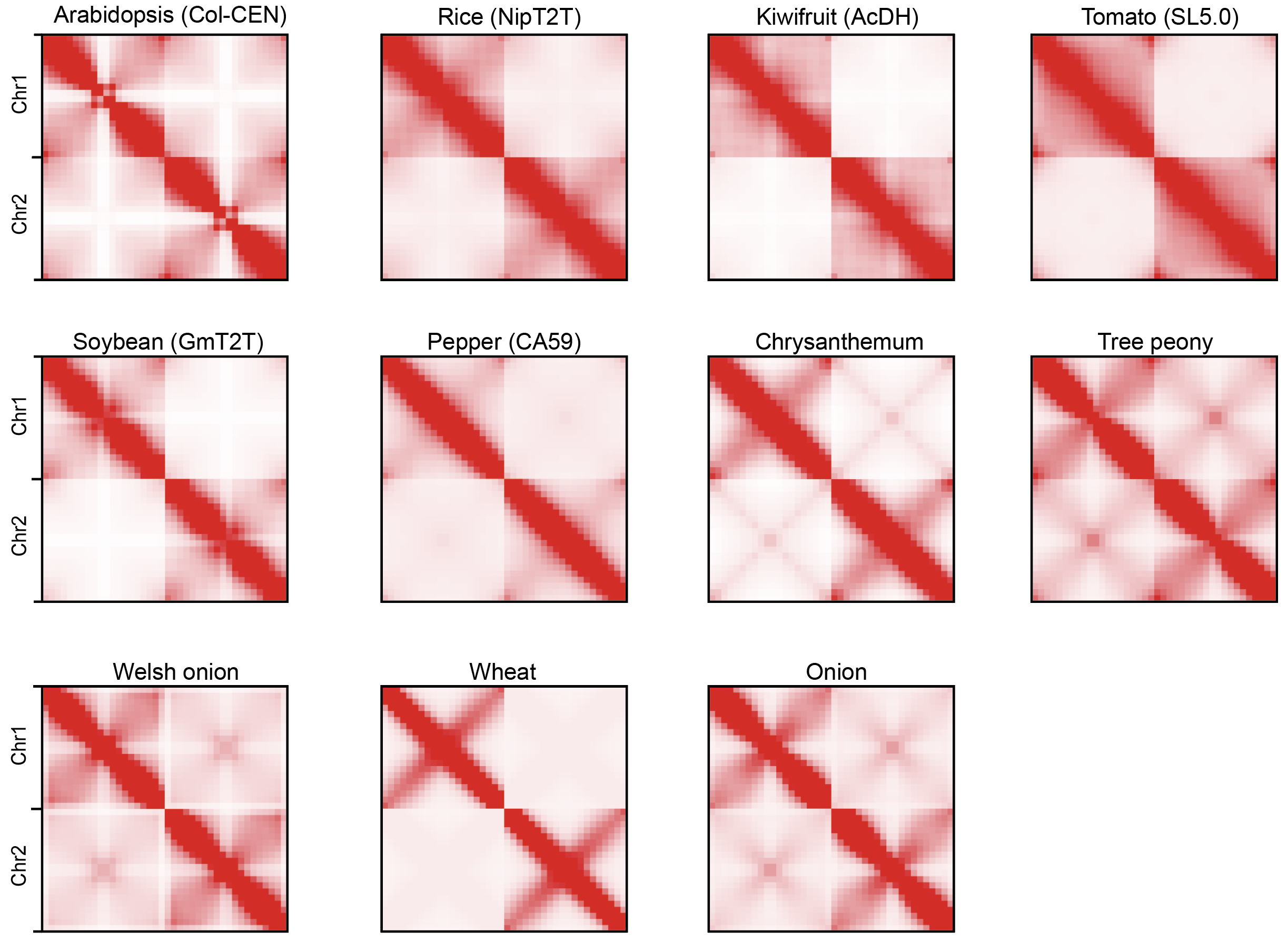


**Figure S2. Aggregate chromosome analysis for the 11 species examined in this study**

Matrices used for axis score calculation. The genome assembly used is marked out. For species with multiple versions of genome assembly, the gapless ones with identified centromeric region are preferred.


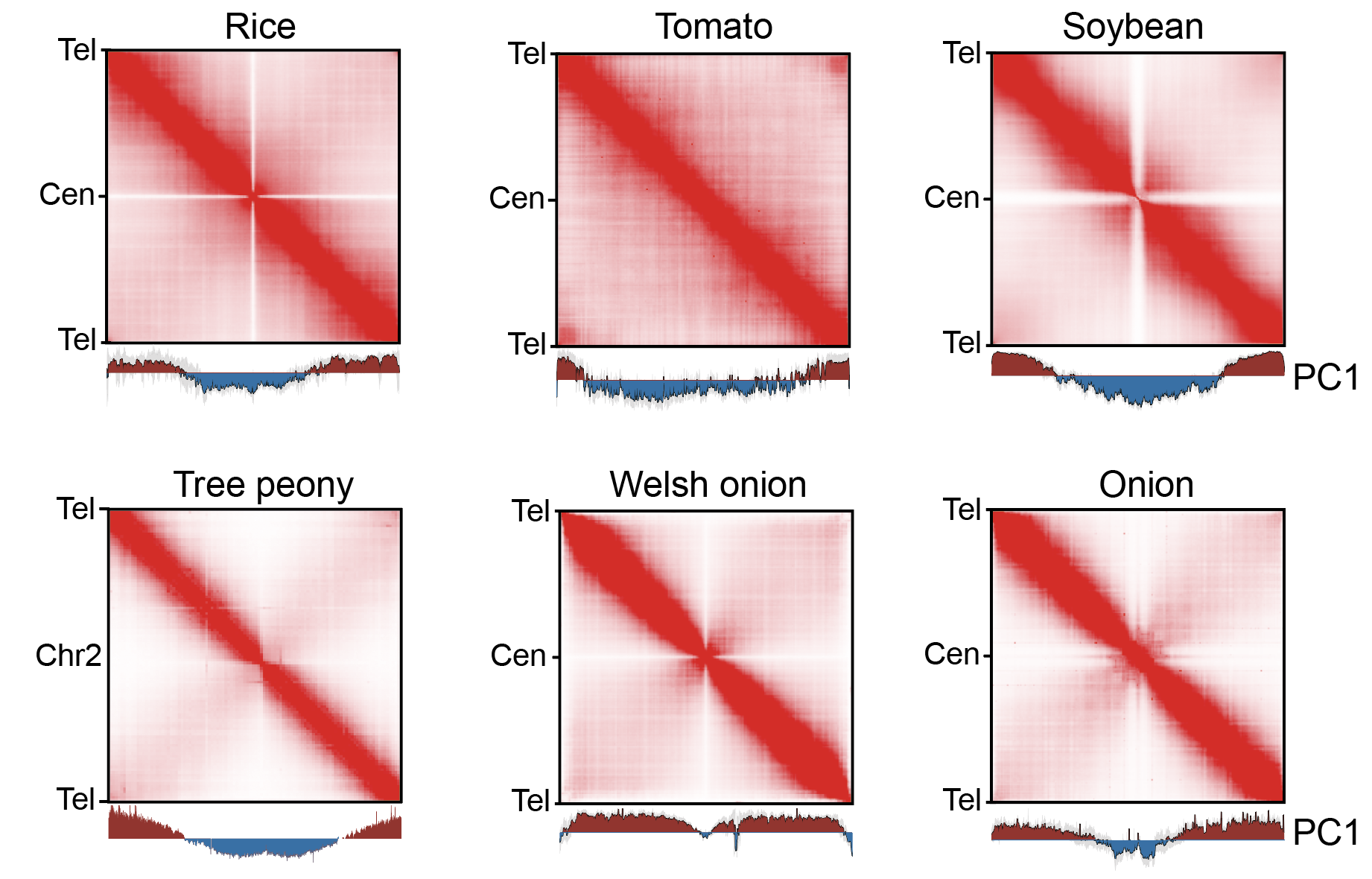


**Figure S3. Aggregated Hi-C matrices with scaled PC1**

For each species, only metacentric chromosomes with known centromeric region are aggregated. PC1 values from each chromosome are scaled and mean value with 95% confidence intervals plotted. Notice: most bioinformatic tools cannot handle giant chromosome (length>2^31bp). For tree peony (2n=10, 10.3 Gb)^14^, only two chromosomes could be aggregated, making PC1 scaling infeasible. Herein, only Chr2 with its own PC1 values is shown.


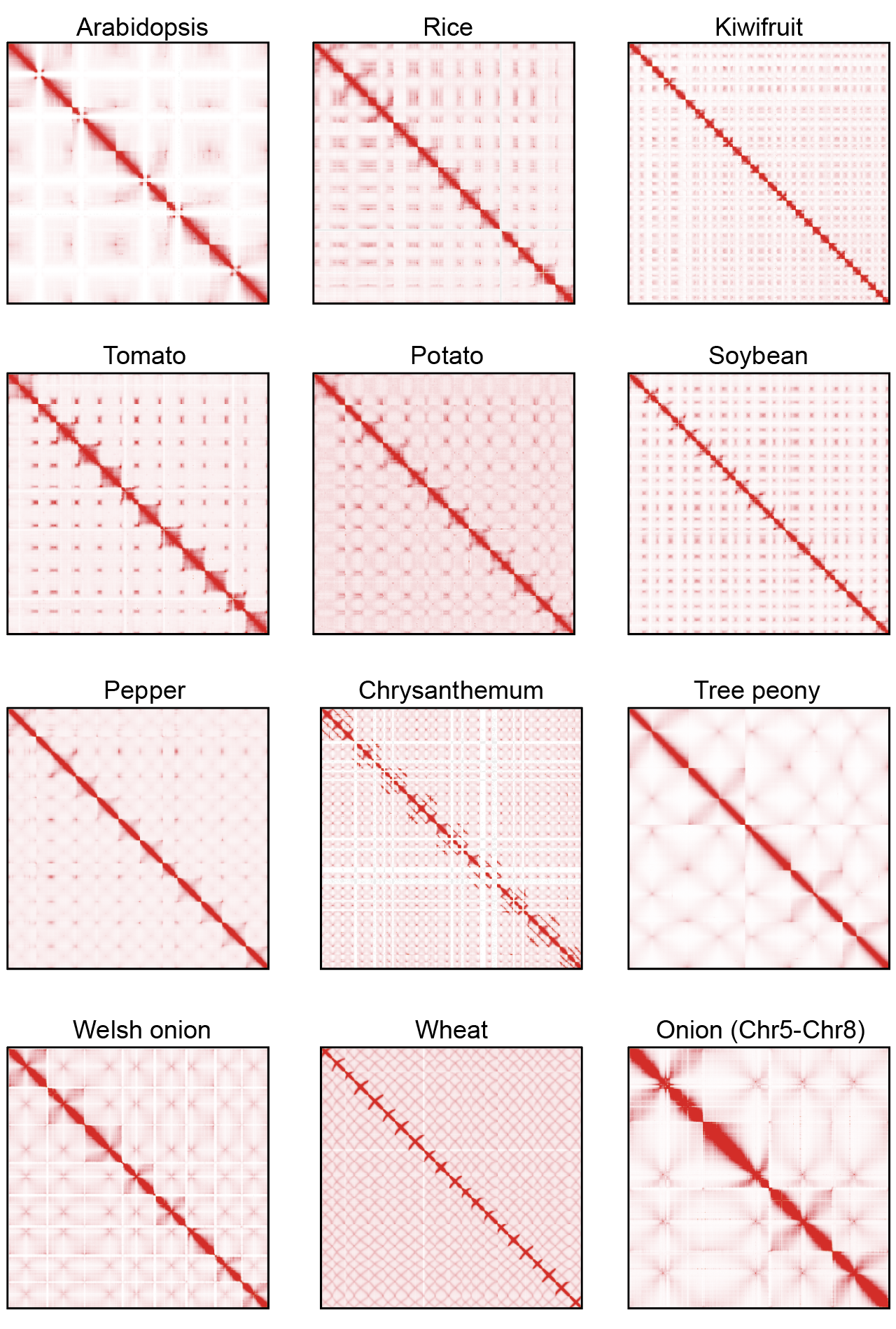


**Figure S4. Raw Hi-C matrices generated in this study**

From top left to bottom right, the genome size increases. For species with small genome size, the telomere clustering is a prominent feature. Whereas species with large genome size tend to form centromere clustering and chromosome axis. For convenience, the onion chromosome 1,2,3,4 longer than 2 Gb are discarded^13^ . For tree peony, the giant chromosome 1,2,4 are each split into two small chromosomes.


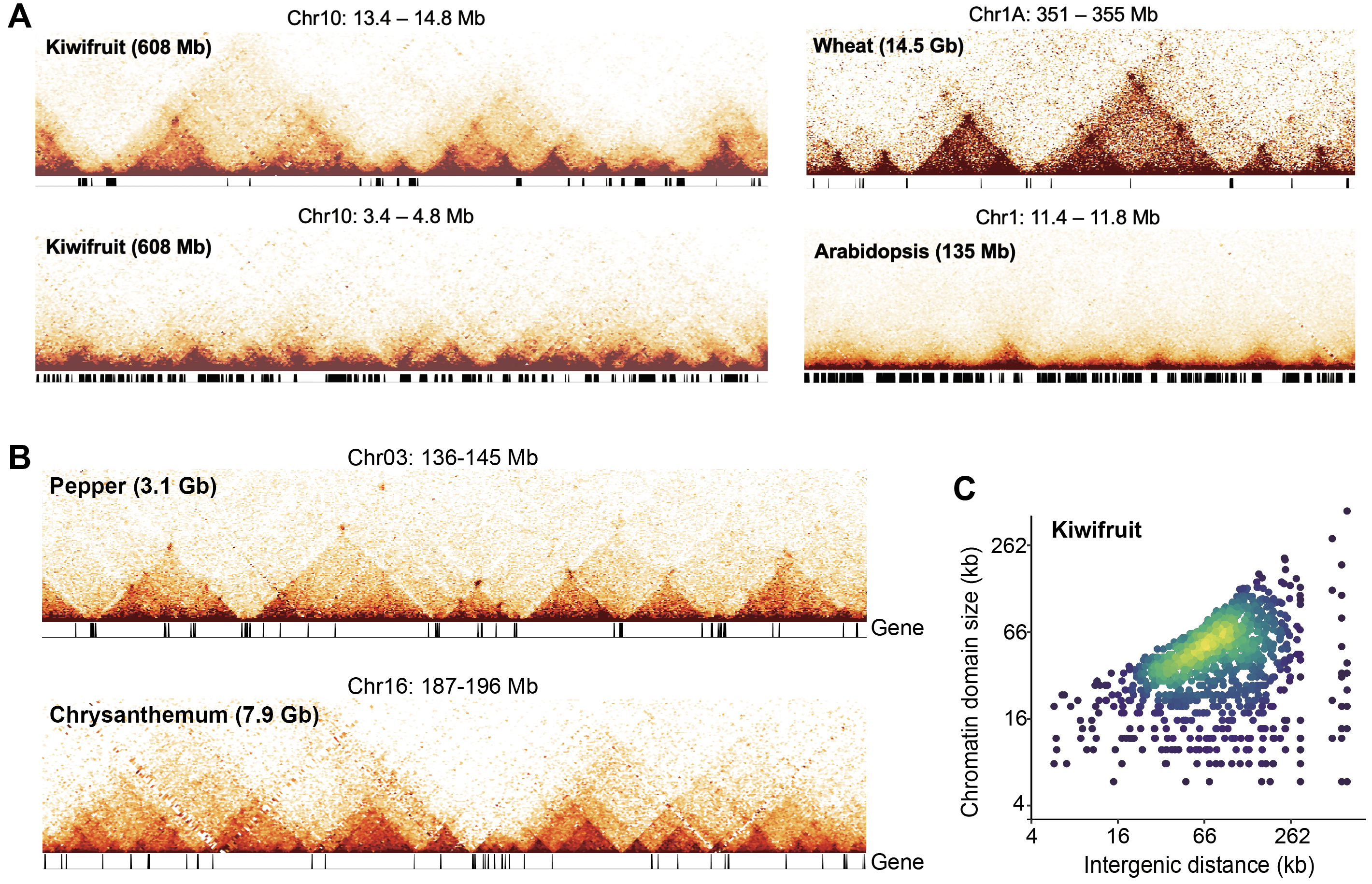


**Figure S5. Intergenic space forms a chromatin domain in gene-sparse region**

**A.** Plant chromatin organization is related to gene density. In kiwifruit gene-sparse region, the intergenic space folds into consecutive chromatin domains sectioned by genes, as we used to see in wheat. Whereas in kiwifruit gene-dense region, just like Arabidopsis, little higher-order structures could be found. **B.** Intergenic region form chromatin domain in pepper and chrysanthemum, too. **C.** Size distribution of chromatin domains as a function of the length of the intergenic space, showing the preferential organization of intergenic region into chromatin domains. Chromatin domains are called in kiwifruit gene-sparse region by cooltools. This tells why species with small intergenic space, such as *Arabidopsis*, seldom exhibits chromatin domains.

**
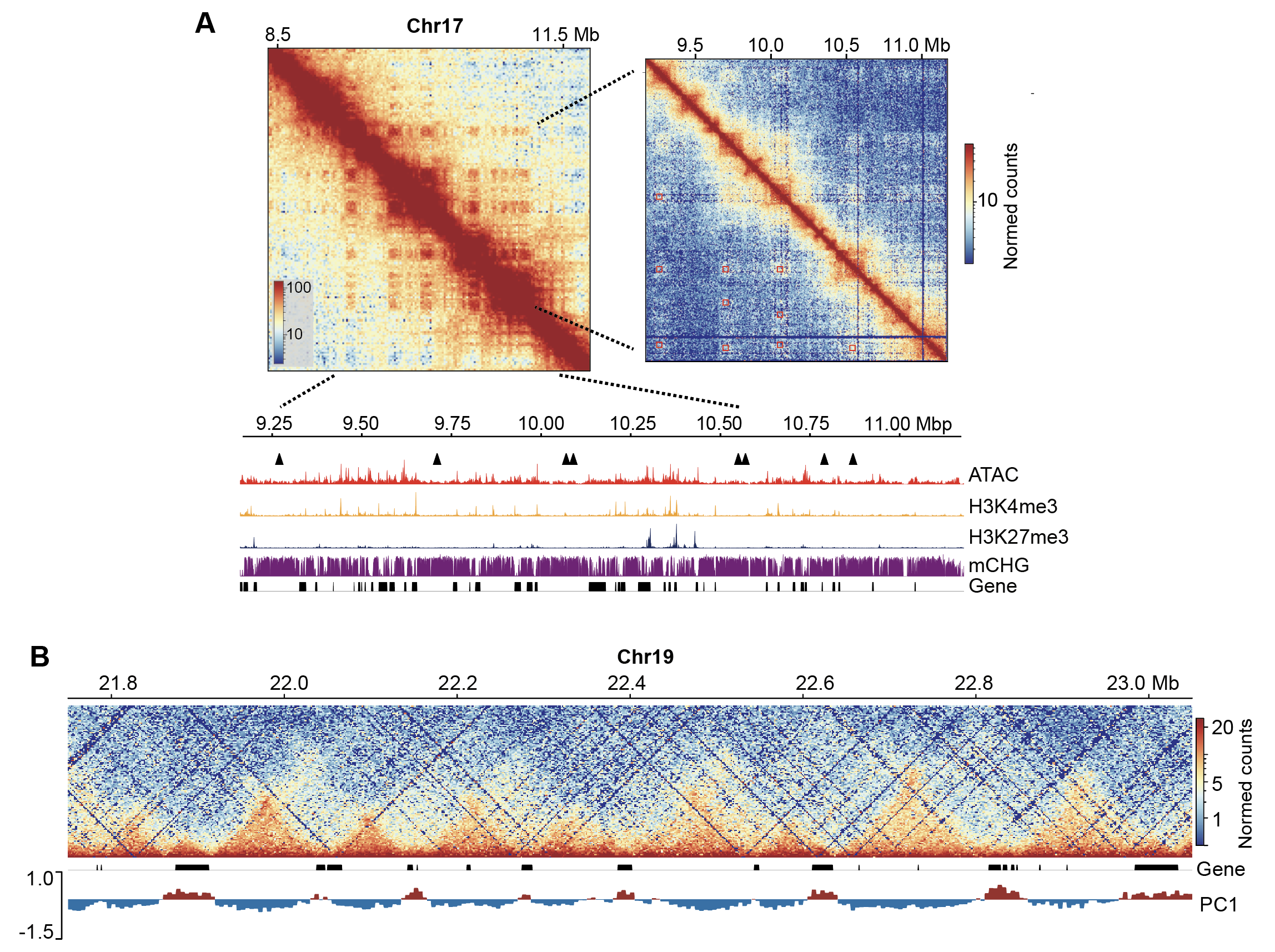
**

**Figure S6. The intergenic chromatin domains are essentially sub-B compartment**

**A**. The contact heatmap of a representative heterochromatic region in kiwifruit Chr17, showing the clustering of intergenic space. Dashed lines indicate enlarged region. Red boxes: peaks called by chromosight under 20 kb resolution. Black triangles: peak coordinates. **B**. The colocalization of sub-B compartment with intergenic chromatin domains. PC1 values are computed by cooltools under 5 kb resolution. Gene bodies are labelled as black rectangles.


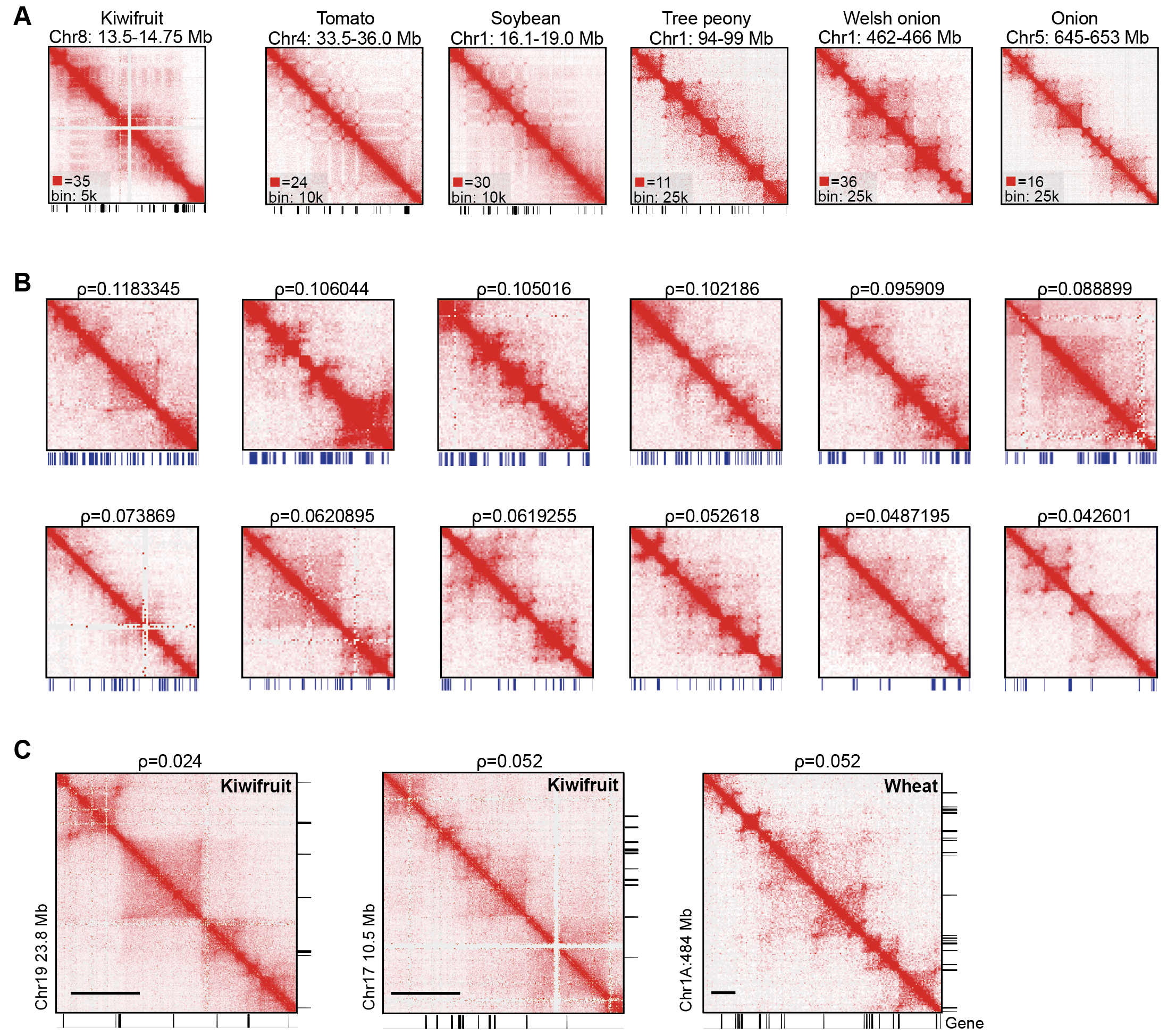


**Figure S7. Gene to gene loops are prevailing in wheat but extinct in kiwifruit genome**

**A.** The inter-domain organization in multiple species. Kiwifruit adopts domain clustering conformation, while gene-to-gene loops emerges in tomato and prevails in onion. **B.** The chromatin contact pattern in wheat, with varying gene densities. Hi-C matrix resolution: 25 kb. Genes were labeled at the bottom. **C**. The chromatin contact pattern in wheat and kiwifruit genomic blocks. Hi-C matrix resolution: 5 kb. Scale bar: 200 kb. Rho: gene densities within. This tells that the presence/absence of GGL in wheat/kiwifruit is robust, regardless of the matrix resolution.


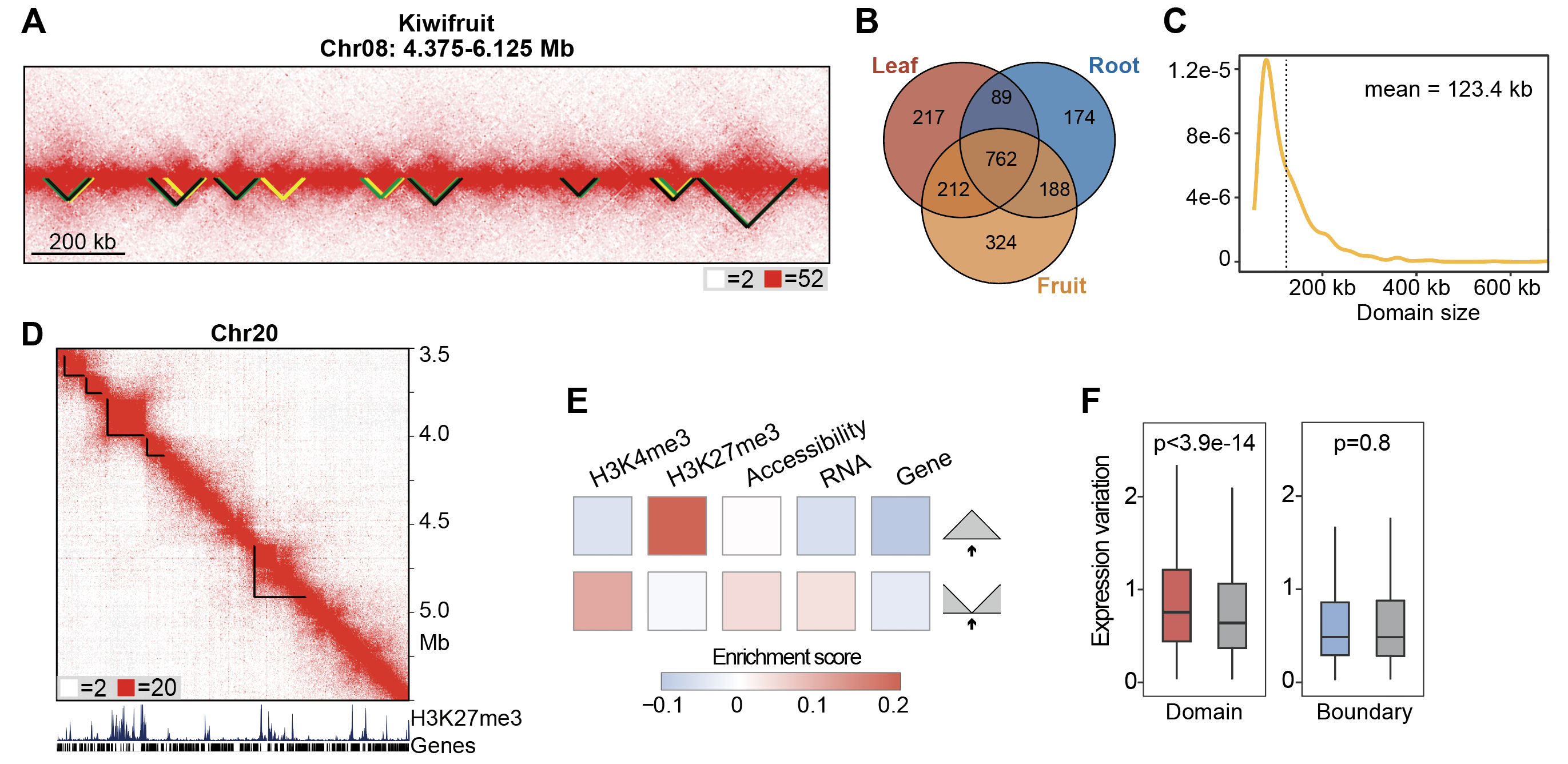


**Figure S8. Identification of chromatin domains in kiwifruit euchromatin region**

**A.** Chromatin domains called by Arrowhead in kiwifruit euchromatin region. Different colors represent domains called in different tissue. Black: root tissue. Yellow: fruit tissue. Green: leaf tissue. **B**. Conservation of domains across tissues. 1310, 1220, 1512 euchromatin domains are identified in leaf, root and fruit, and a vast majority (1250) is shared in at least two tissues. **C.** The size distribution of 1250 chromatin domains in kiwifruit genome. Dashed line indicates mean value. **D.** A contact map of euchromatin region in kiwifruit root tissue. Domains called by arrowhead are marked by black triangle frames. The domain localization coincides with H3K27me3 deposition. **E**. Feature enrichment on domains and domain boundaries. Positive values indicate enrichment and negative values indicate depletion compared against random positions. **F**. Box plots showing the deviation of gene expression level in three tissues. The random intervals are the same with (C). P-values are derived from Wilcoxon test. Domains here are enriched for H3K27me3 and embed genes that have undergone the most drastic change of expression level among tissues.


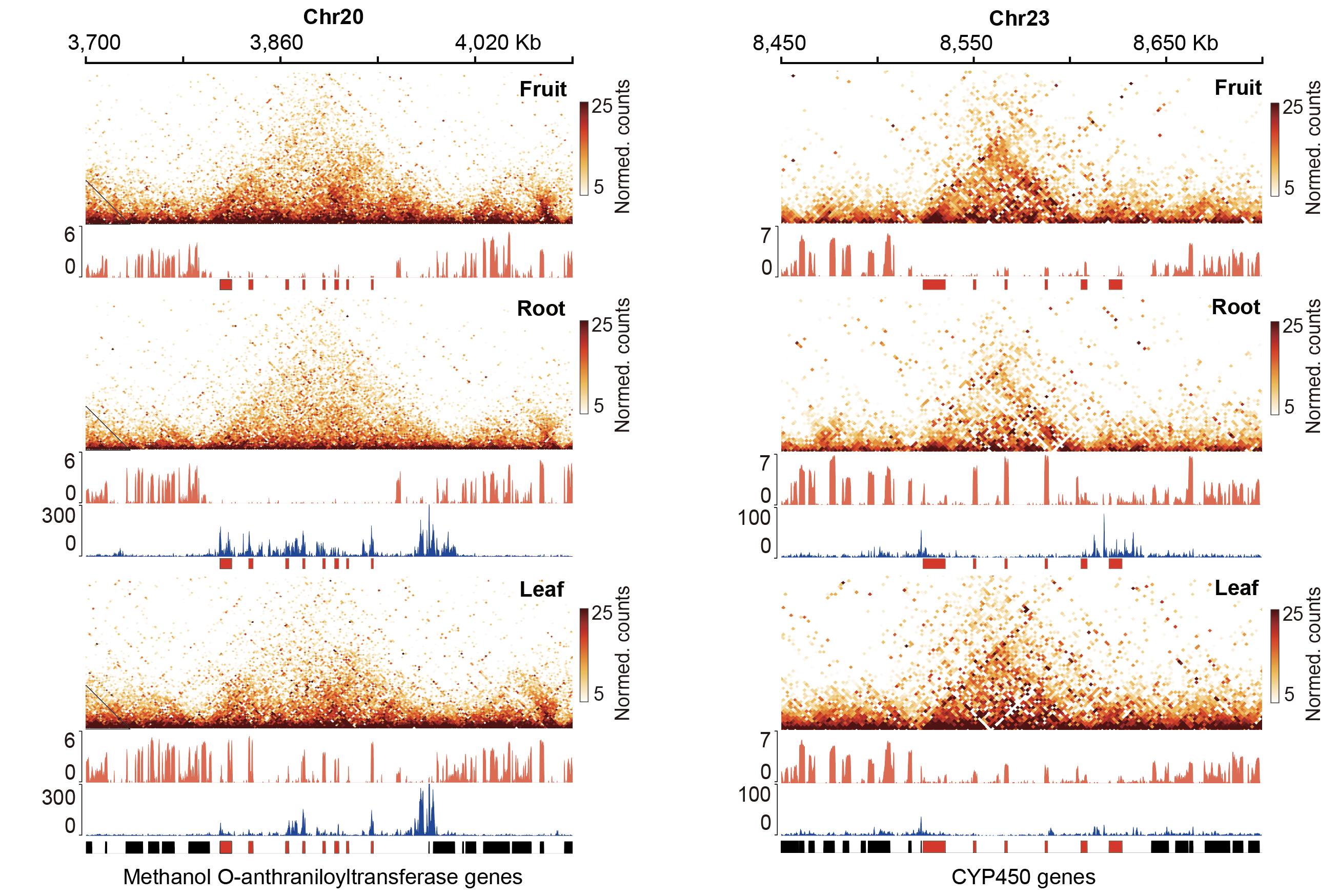


**Figure S9. Colocalization of tandem duplicate gene clusters with chromatin domains**

Local contact heatmap with corresponding H3K27me3 deposition and transcripts abundance in three tissue samples. Rectangles labeled red were tandem duplicate genes. The Hi-C matrices from different tissues have been scaled to the same read coverage.


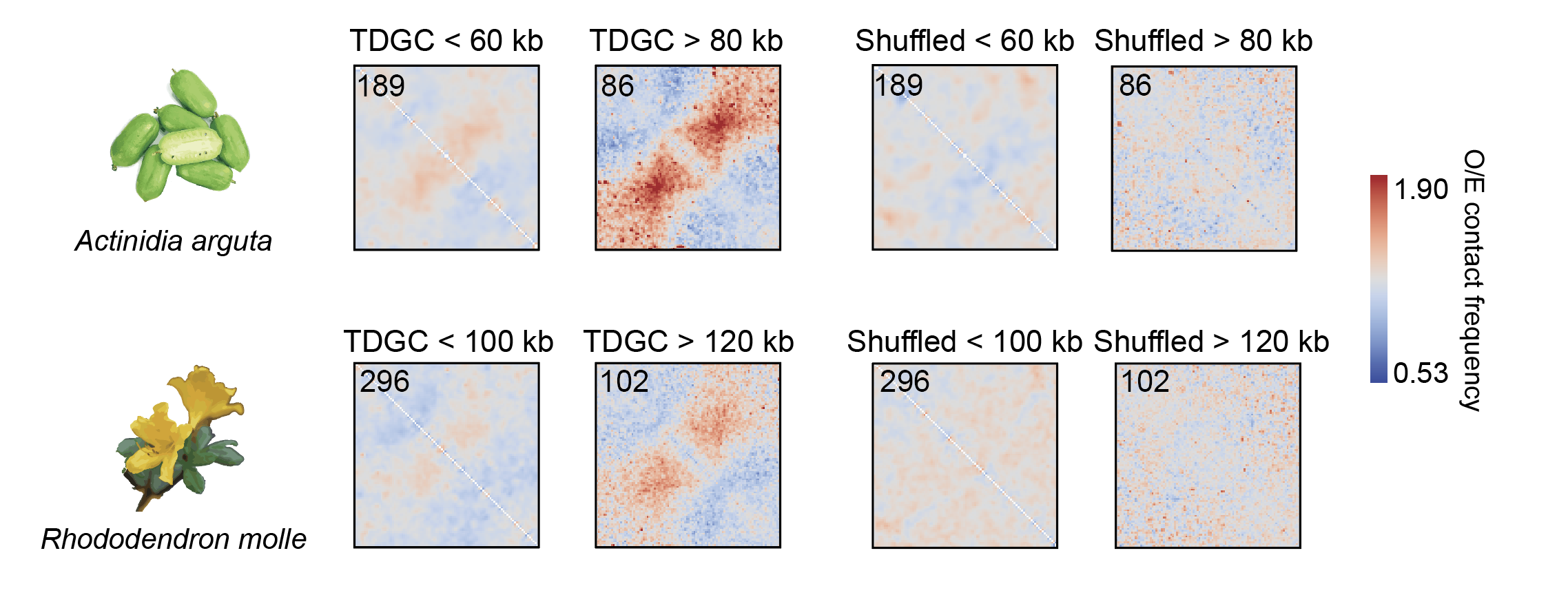
**Figure S10. Tandem duplicate gene clusters preferentially fold into chromatin domains**

Pile-up analysis for TDGCs with small and large spanning length in *A. arguta* and *R. molle*, respectively. Random genomic regions were generated by ‘bedtools shuffle’.

**
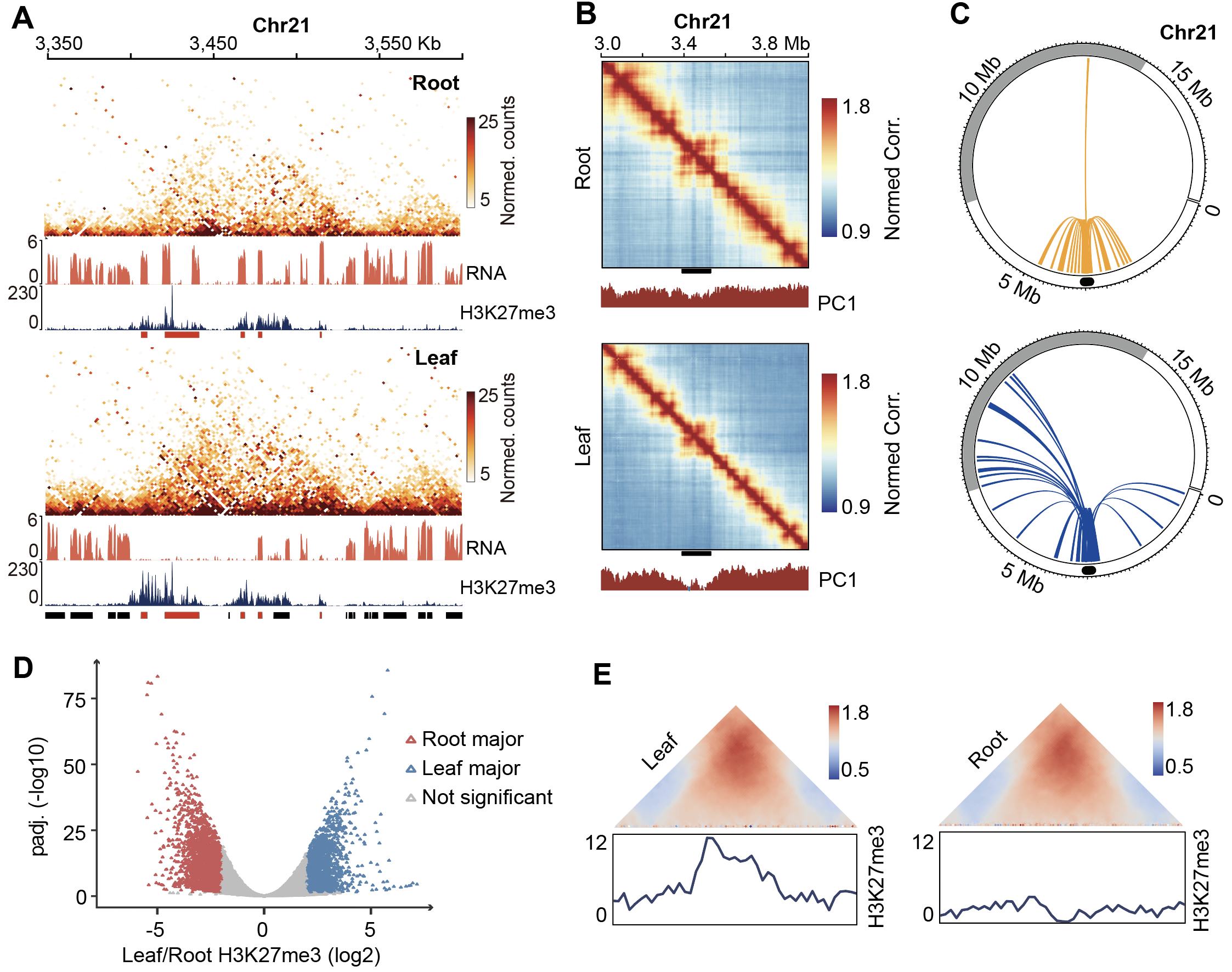
**

**Figure S11. The interrelation between H3K27me3 and TAD-like domains**

**A**. The TAD-like domain embedding *patatin* gene cluster. *patatin* genes are colored red. Hi-C matrices and RNA/H3K27me3 abundance from two tissue samples have been scaled to the same read coverage. **B**. Pearson correlation matrices transformed from distance-normalized Hi-C matrices. The negative (blue) PC1 values indicate repressive compartment while red ones indicate permissive compartment. **C**. Circos plots showing differential intrachromosomal interactions of *patatin* cluster in leaves and roots. Chromosome arms are shown in white, central B compartments are shown in gray, and the *patatin* cluster is shown in black. **D**. Volcano plots showing genomic regions with differential H3K27me3 deposition between kiwifruit leaf and root tissues. **E**. Pile-up plots for 297 domain regions with differential H3K27me3 deposition between leaf and root.


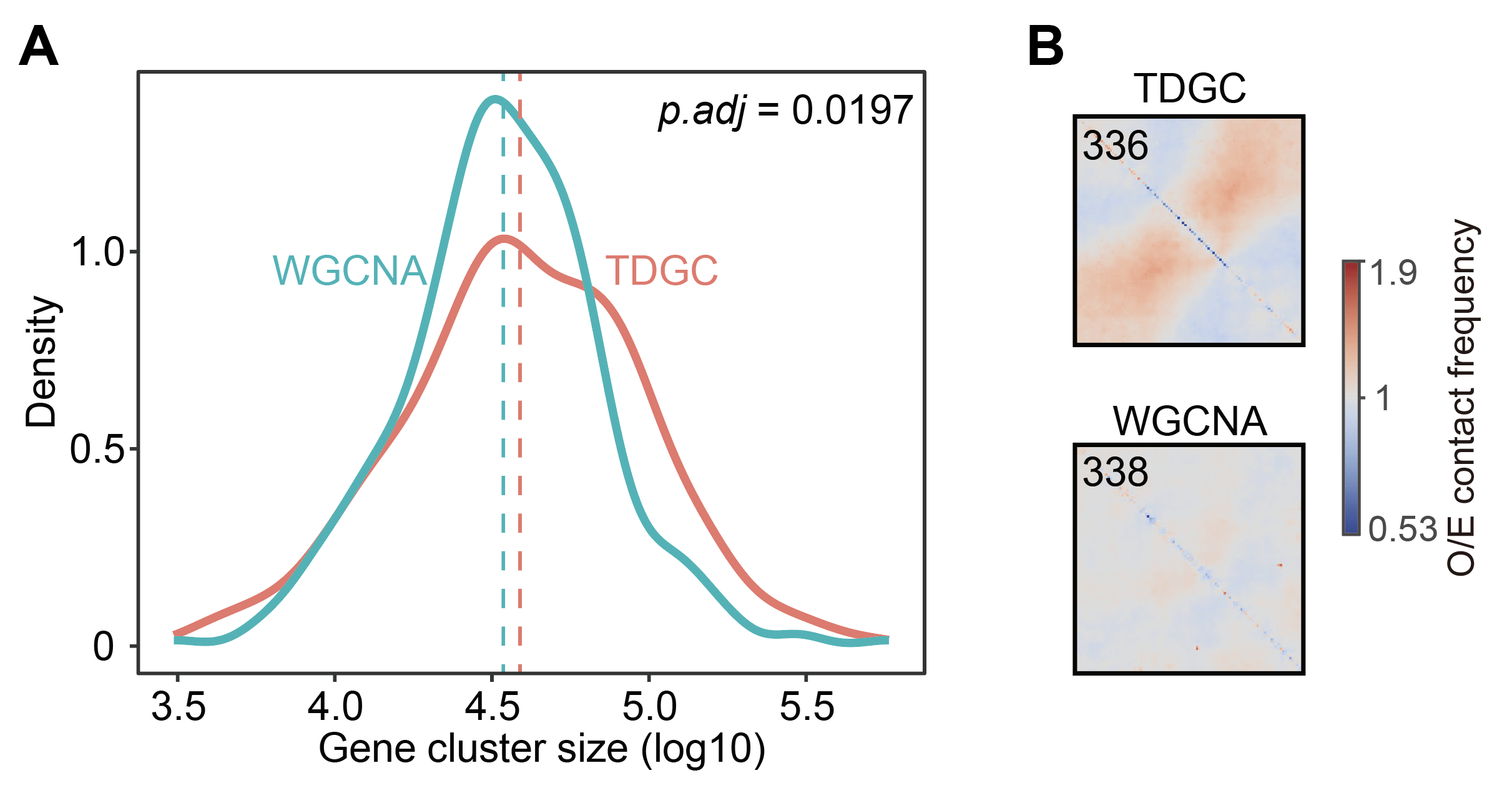


**Figure S12. Co-expressing gene arrays do not form chromatin domains**

**A.** The size distribution of tandem duplicate gene clusters (TDGCs) and co-expressing gene arrays identified by WGCNA^15^. The mean values are indicated by dashed lines. P values are derived from t-test under Bonferroni correction, indicating the difference between the two curves. The size distributions of TDGC and co-expressing gene arrays are quite similar. **B.** Pile-up analysis for TDGCs and co-expressing gene arrays (for simplicity, labelled as WGCNA). Co-expressing gene arrays are incapable of forming a chromatin domain, and such incompetence is not due to the gene array size.


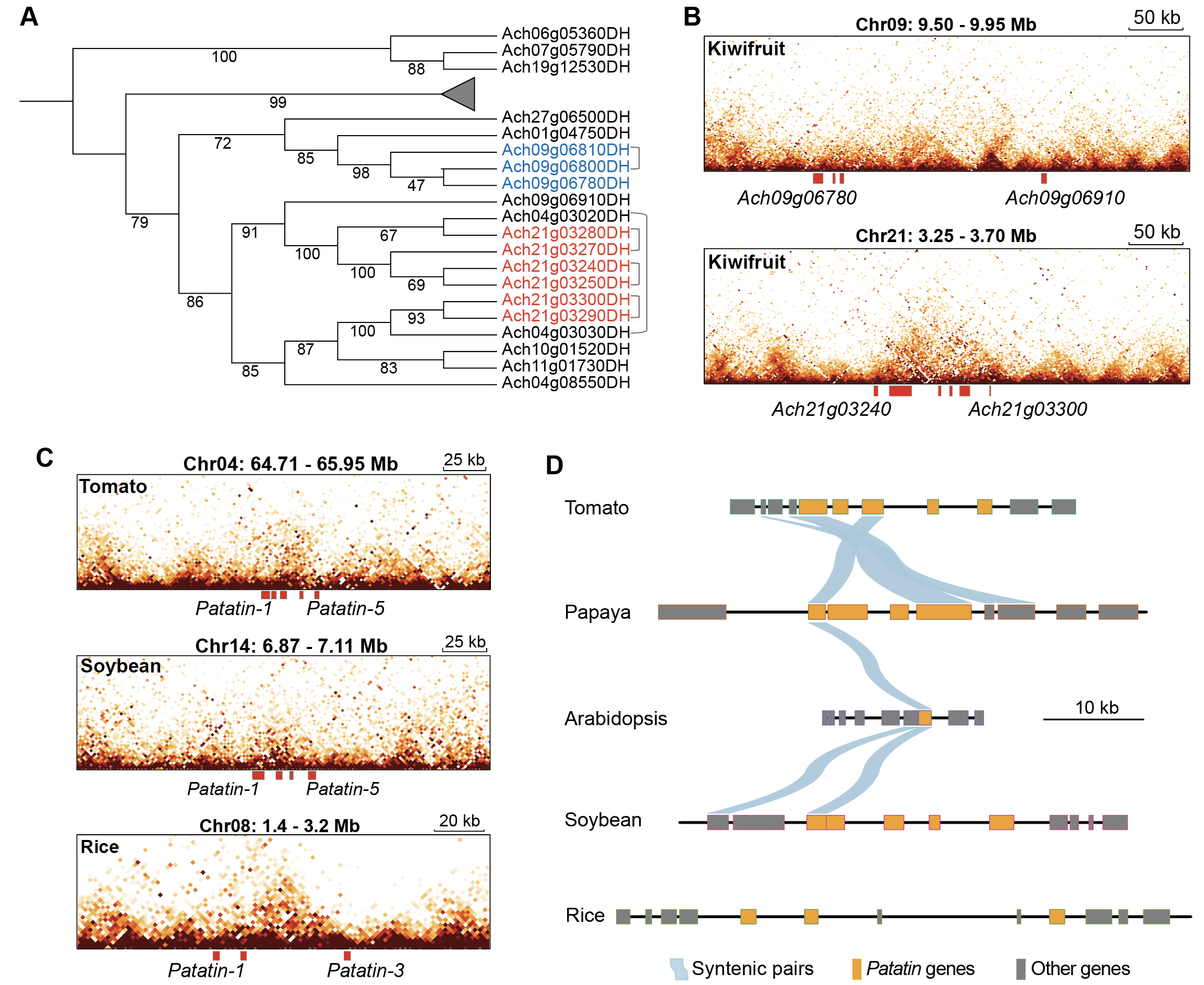


**Figure S13. The local chromatin organization on patatin gene cluster across species**

**A**. The Neighbor-joining tree of kiwifruit *patatin* genes (percent bootstrap support indicated). Genes labelled blue or red are defined as tandem duplicate gene clusters, with gene copy number >=3 and intervening non-homologs <=5. Genes connected by lines are tandem duplicates identified by MCScanX. **B.** The local chromatin contact map for the two patatin gene clusters in kiwifruit, as labelled in (A). The smaller patatin gene cluster cannot form a TAD-like domain. Resolution for Hi-C matrix: 2 kb. **C.** The local chromatin contact map for patatin gene clusters in tomato, soybean and rice. In tomato and soybean, the gene clusters are too small to form a chromatin domain. **D.** Microsyntenic segments centered around *patatin* genes. The segment length is of the same scale. The patatin gene cluster in rice shows no syntenic relation with other eudicots, where a chromatin domain still comes into shape, as shown in (C).


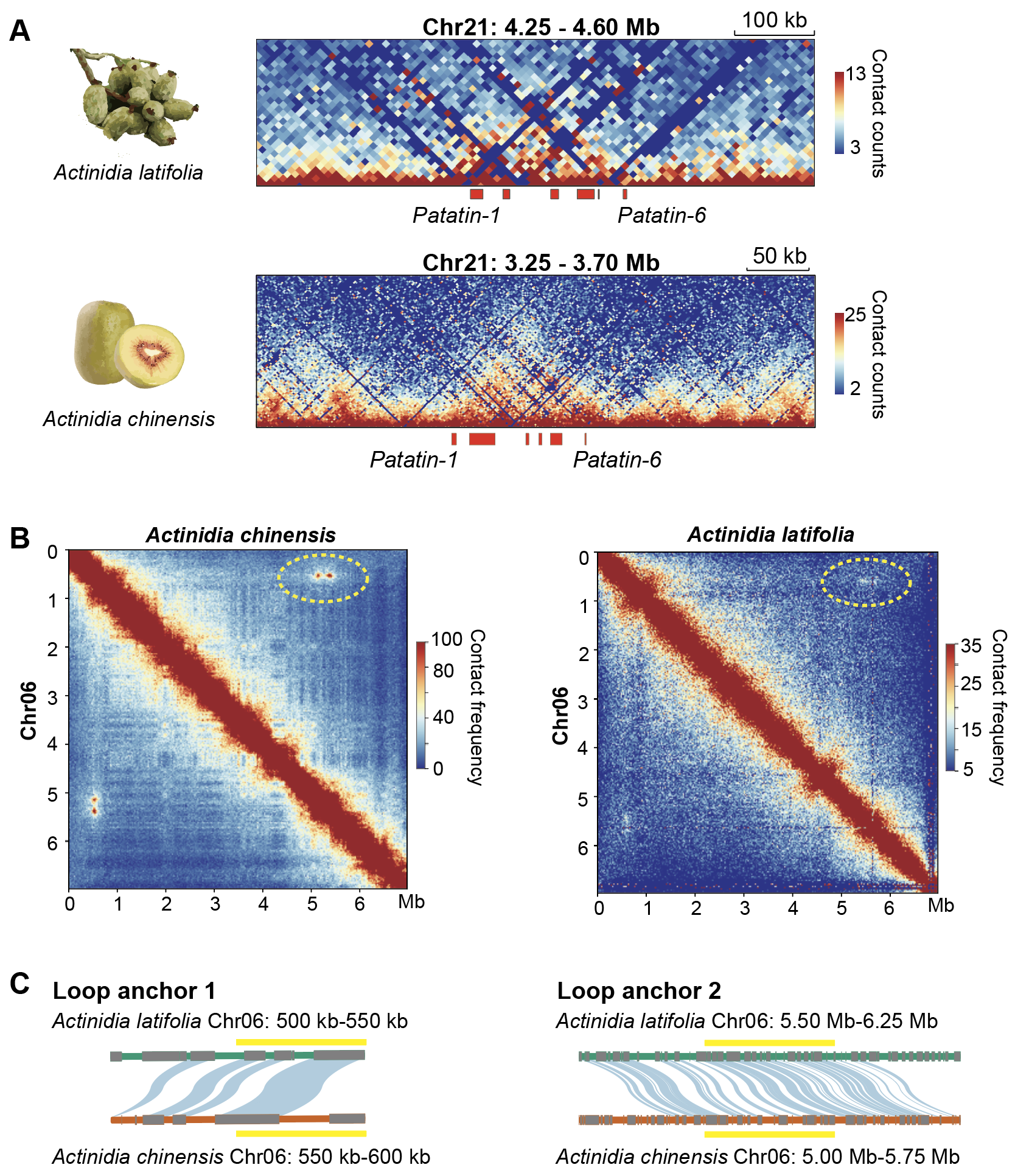


**Figure S14. Conservation of 3D structure in syntenic blocks between *A. chinensis and A. latifolia***

**A.** The TAD-like domain embedding *patatin* gene cluster in *A. latifolia* and *A. chinensis*. *Patatin* genes are colored red. Hi-C matrix resolution for *A. latifolia* and *A. chinensis* are 5 kb and 2 kb, respectively. **B.** The long-range chromatin loops in *A. chinensis* and *A. latifolia*. **C.** The loop anchor regions share synteny between the two species. Yellow line: loop anchors called by chromosight under 25 kb resolution.


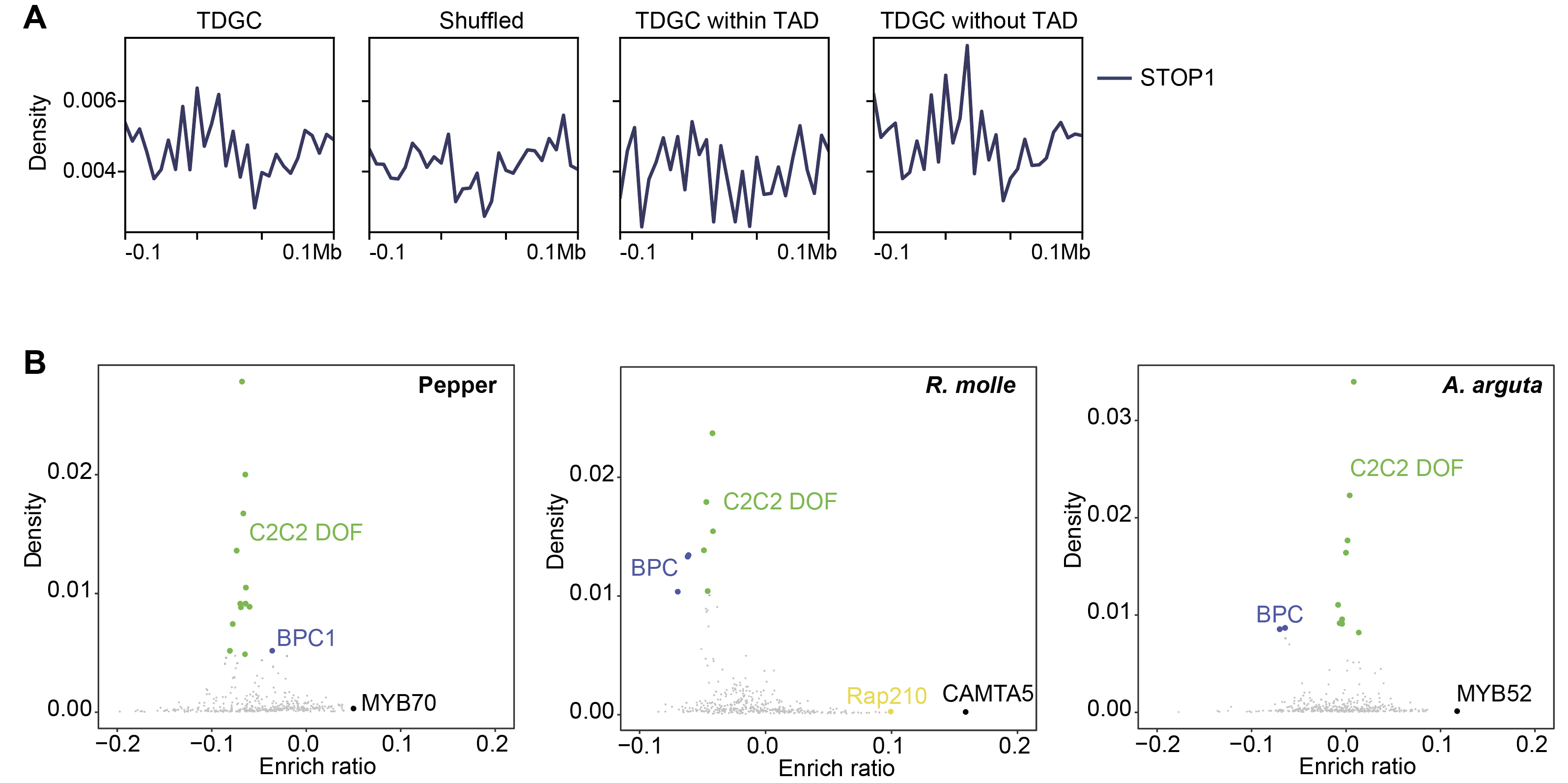


**Figure S15. Motif enrichment in tandem duplicate gene clusters in multiple species**

**A.** The density of STOP1 consensus binding sites across TDGC, TAD-like domain and random genomic regions in kiwifruit. STOP1 extensively presents in TDGCs without TAD-like domain, indicating that it is not the authentic domain mediator. **B.** Motif enrichment analysis in TDGCs in multiple species. TFs including MYB70, CAMTA5 and MYB52 are enriched in different species. Fold enrichment is calculated as the ratio of motif density in TDGC over that in 40 kb flanking genomic regions. Motif density is calculated for 1 kb genomic bins.


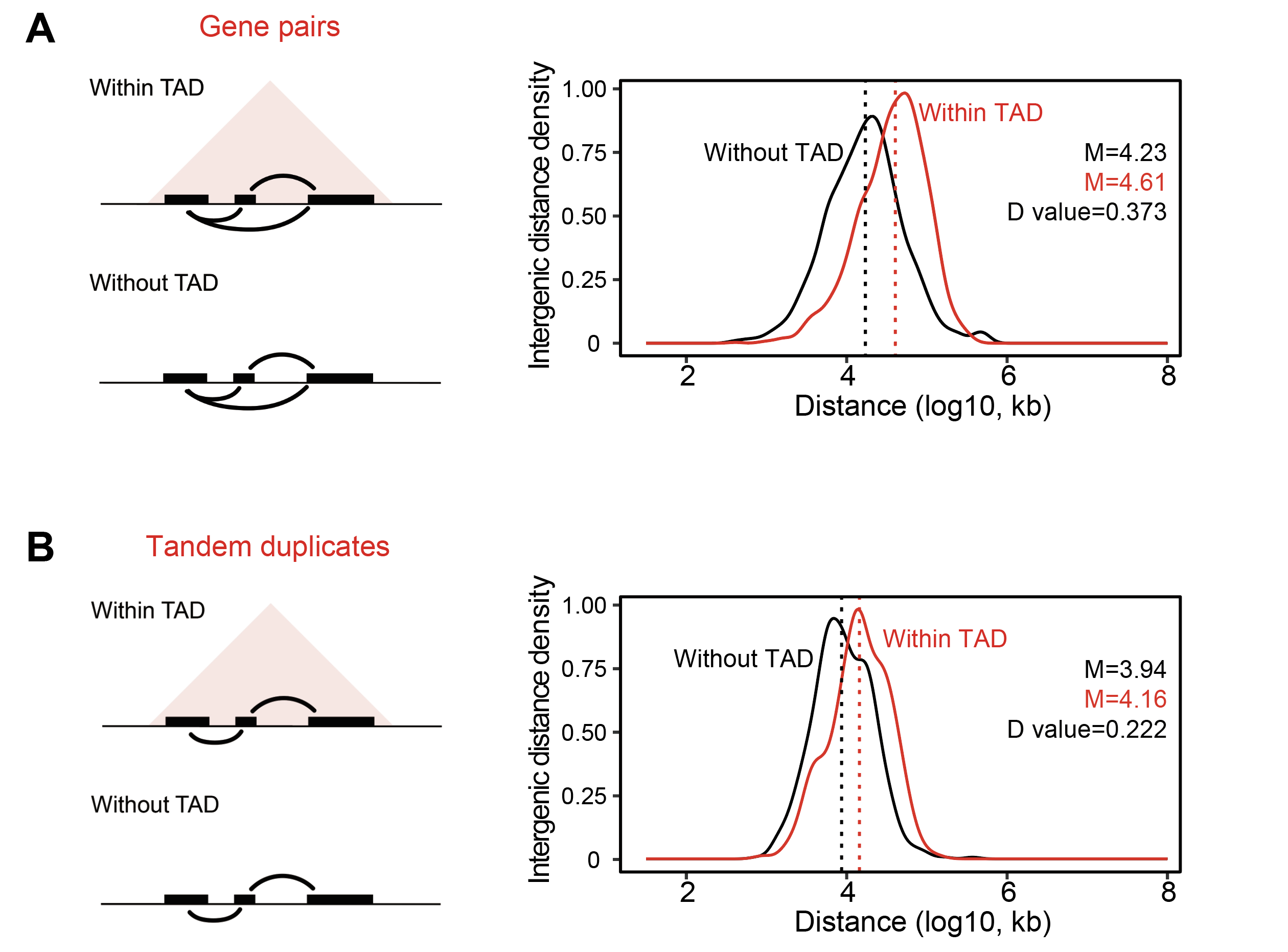


**Figure S16. The distribution of intergenic distance for duplicate pairs and tandem duplicates within or without TAD**

**A.** For gene pairs within and without TAD, the distribution of their genomic distance is plotted. M: median value. D: Difference in median value. Dashed line indicates the median value. **B.** For tandem duplicates within and without TAD, the distribution of their genomic distance is plotted. The deviation on the distribution is controlled.


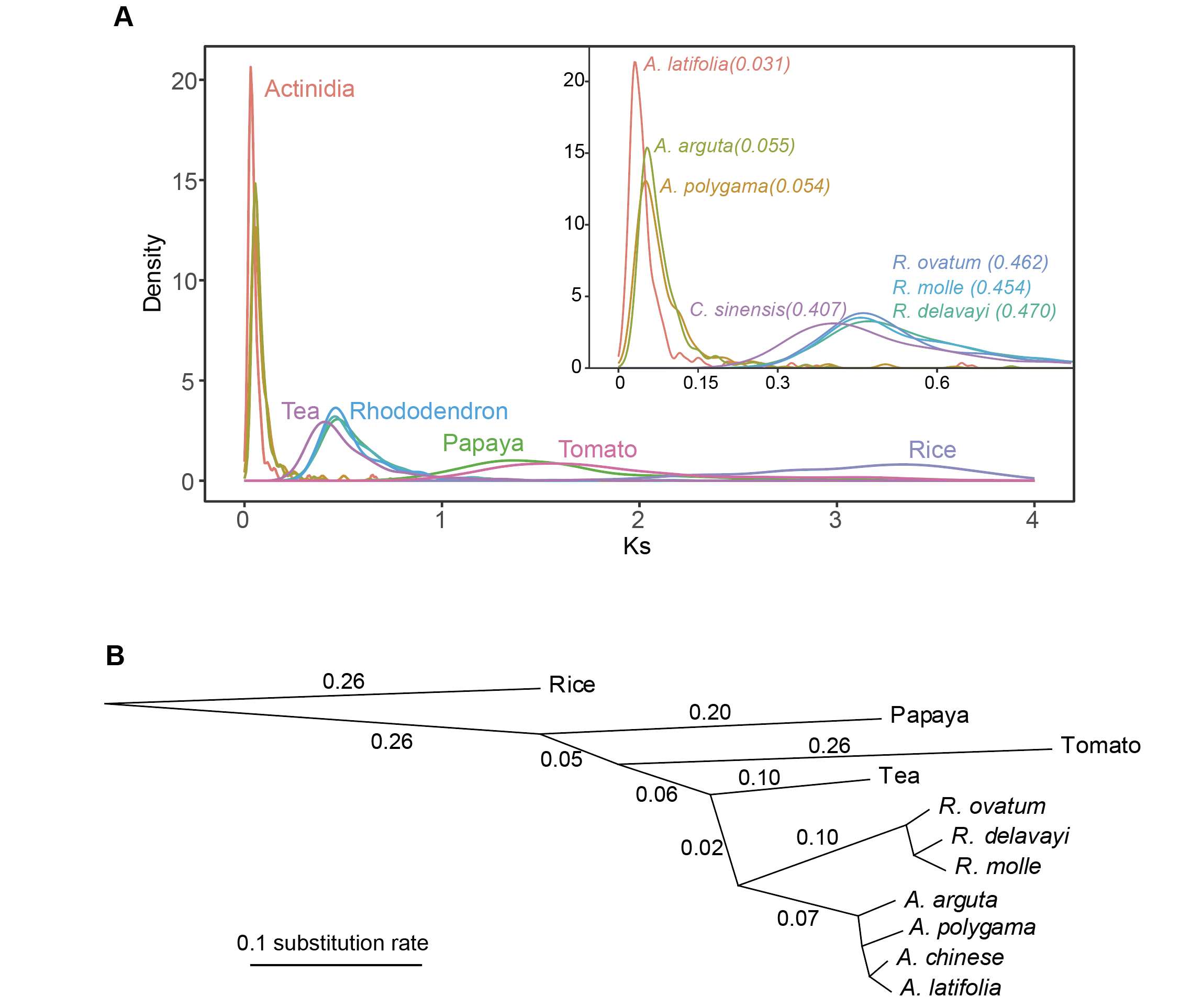


**Figure S17. Phylogenetic analysis for 11 angiosperms, showing that the interval (0.15<Ks<0.3) happens to be the split between Actinidiaceae and Ericaceae**

**A.** Synonymous substitution rate (Ks) distribution of single copy orthologs identified between *Actinidia chinensis* and other 10 species. Numbers in the brackets are the weighted average Ks for ortholog pairs between *Actinidia* *chinensis* and other organisms. **B**. Nucleotide substitution tree of 11 species at 353 single copy orthologs. Branch length: substitution rate.


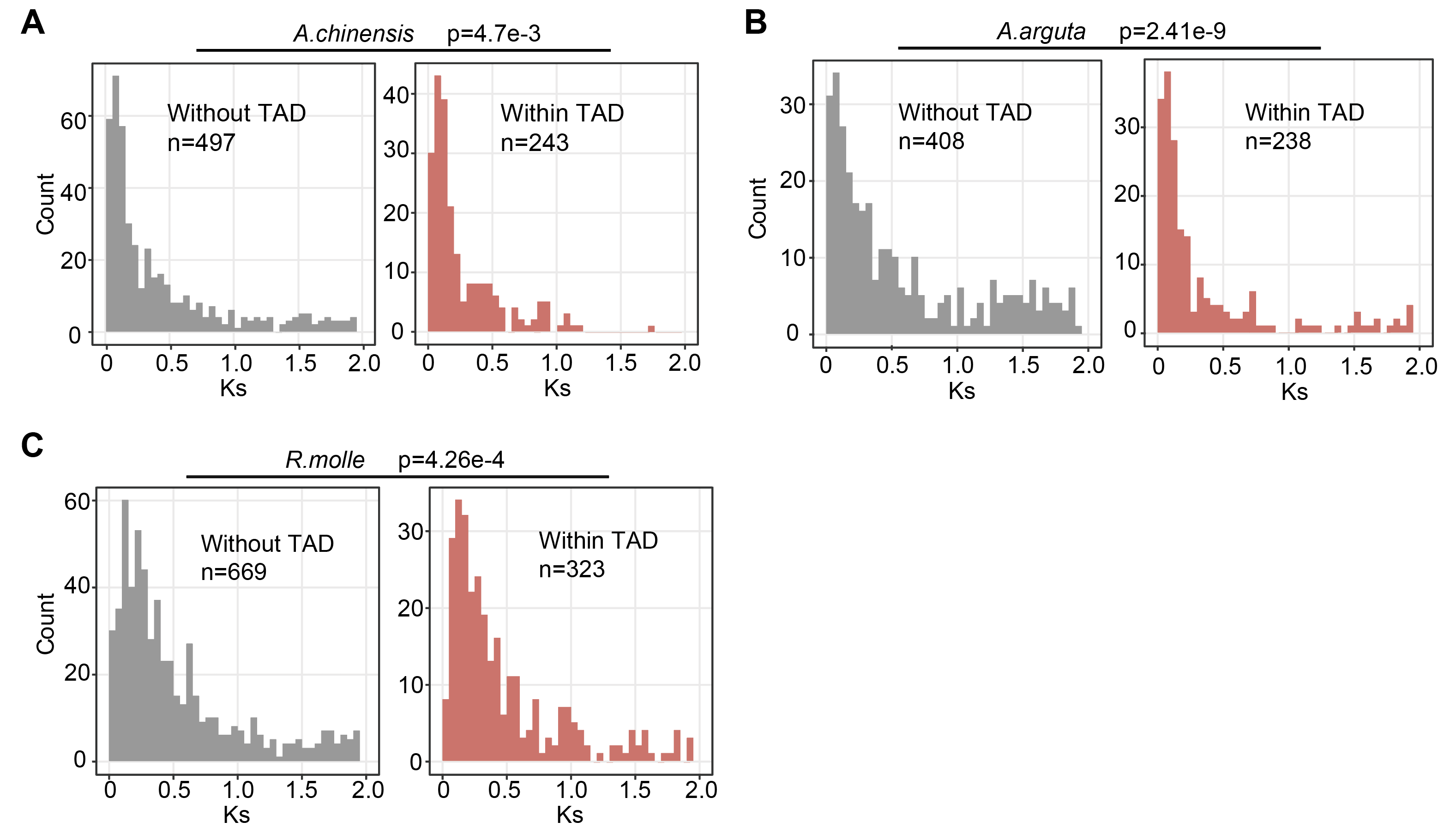


**Figure S18. More young duplicates exist within TAD-like domain in *Actinidia***

Frequency distributions of pairs of tandem duplicates as a function of the number of silent substitutions per silent site in species including (**A**) *A. chinensis*, (**B**) *A. arguta* and (**C**) *R. molle.*


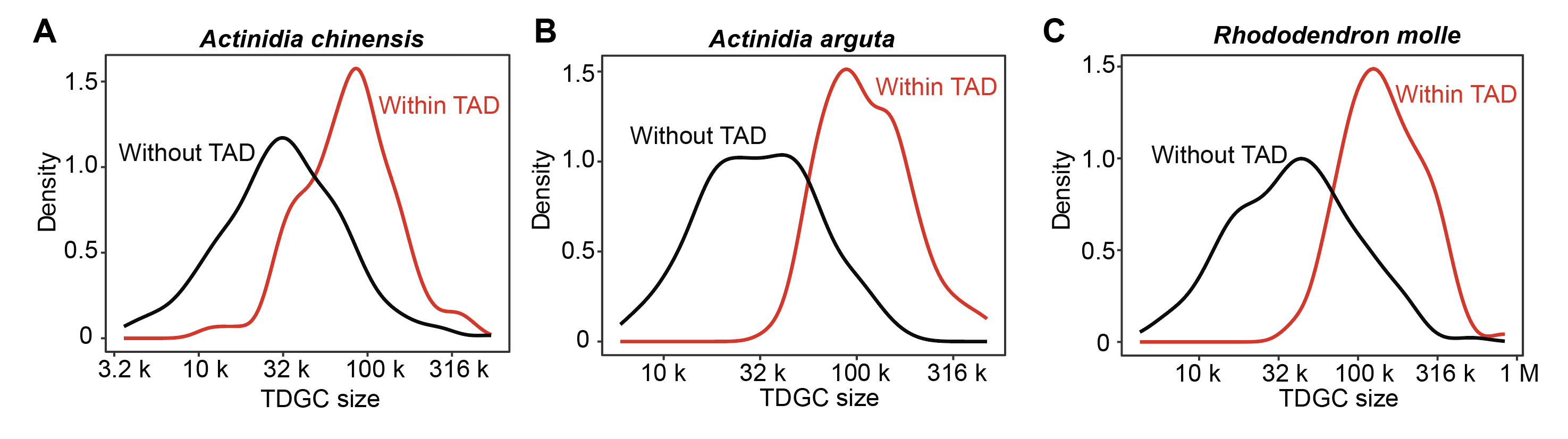


**Figure S19. TDGCs within TAD-like domains are substantially larger**

The size distribution of TDGCs within and without TAD-like domain in multiple species including (**A**) *A. chinensis*, (**B**) *A. arguta* and (**C**) *R. molle.*


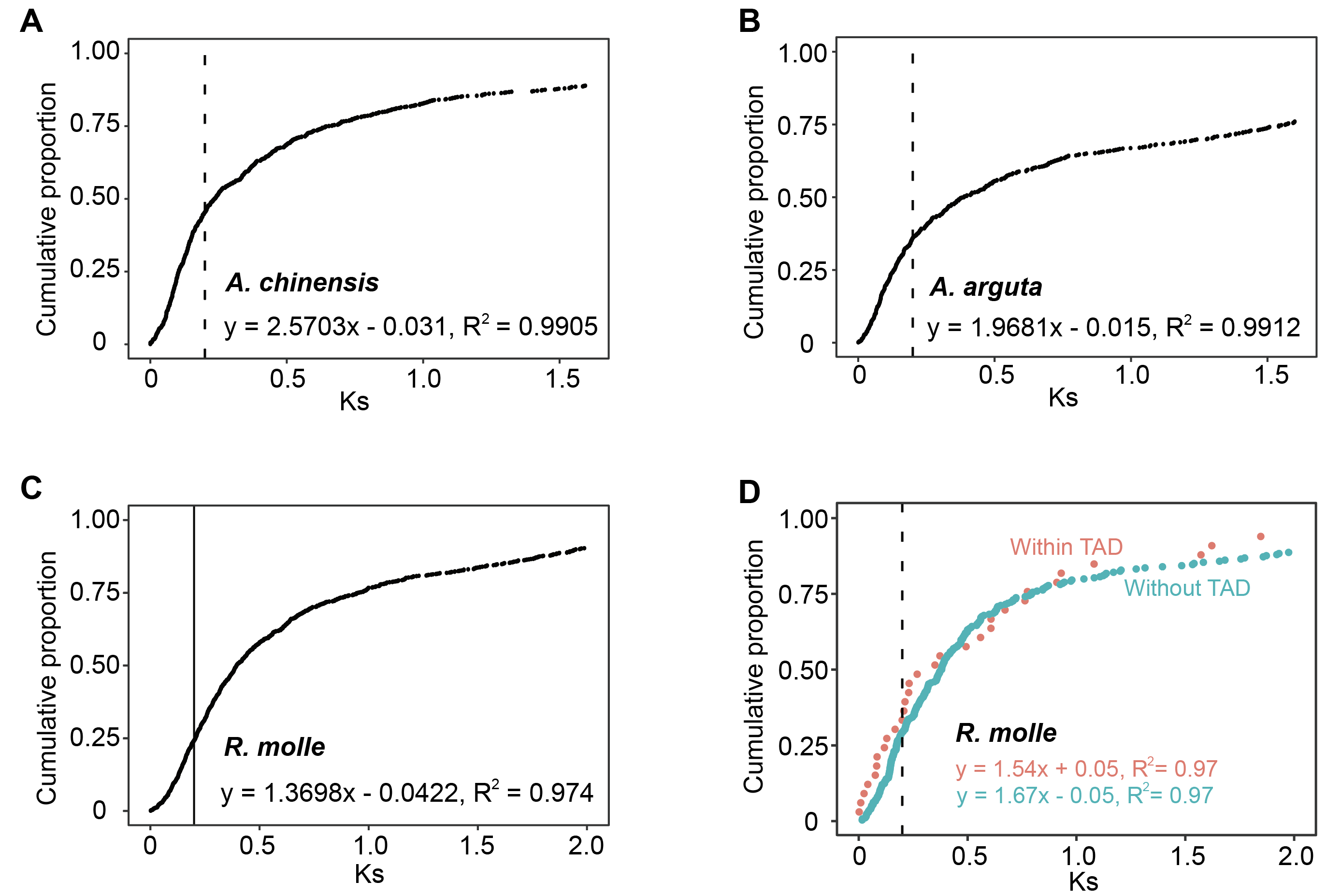


**Figure S20. The duplication frequency varies greatly among species**

The cumulative distribution of tandem duplicates in multiple species including (**A**) *A. chinensis*, (**B**) *A. arguta* and (**C**) *R. molle.* The slope of the linear function for each curve with Ks<=0.2 indicates the duplication rate. D. Cumulative distribution of tandem duplicates in three-copy TDGCs in *R. molle*, as a function of Ks.


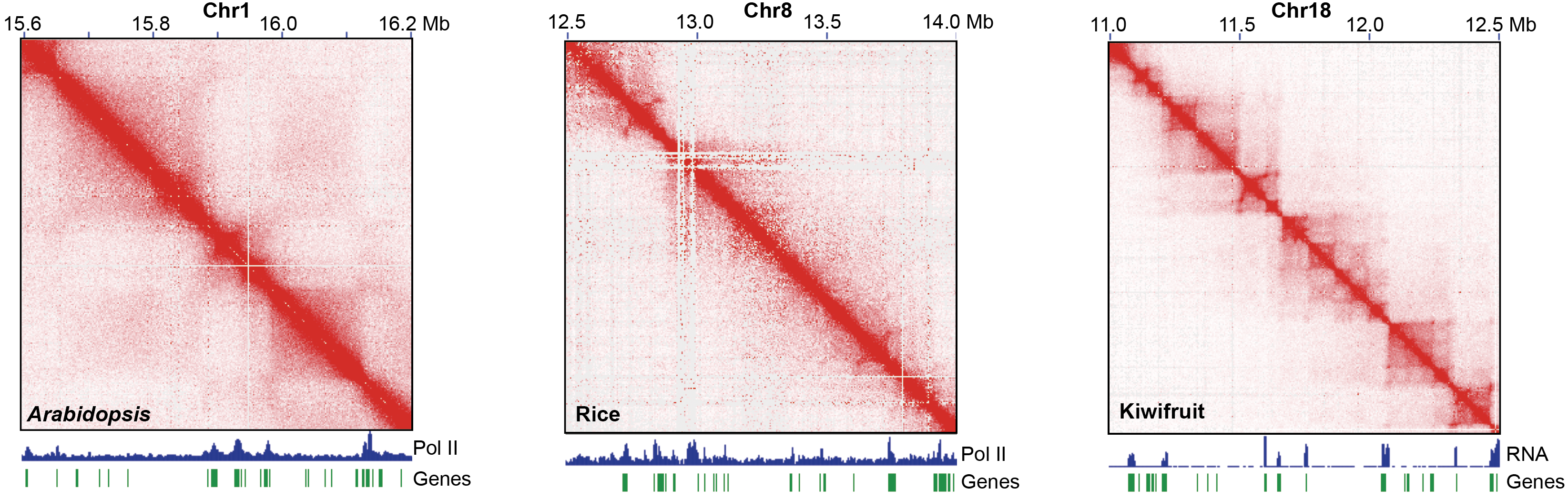


**Figure S21. Species with small genome size lacking GGL conformation**

Hi-C matrices of gene-sparse region in *Arabidopsis*, rice and kiwifruit, respectively. The occupancy profile of RNA polymerase II in *Arabidopsis* and rice is downloaded from Sun *et al.*^16^ and Zhao *et al.*^17^

**Reference**

1. Ramirez, F., Dundar, F., Diehl, S., Gruning, B.A. & Manke, T. deepTools: a flexible platform for exploring deep-sequencing data. *Nucleic Acids Res* **42**, W187-91 (2014).

2. Zhang, Y. *et al.* Model-based analysis of ChIP-Seq (MACS). *Genome Biol* **9**, R137 (2008).

3. Yao, X. *et al.* The first complete chloroplast genome sequences in Actinidiaceae: genome structure and comparative analysis. *PloS one* **10**, e0129347 (2015).

4. Schultz, M.D. *et al.* Human body epigenome maps reveal noncanonical DNA methylation variation. *Nature* **523**, 212-6 (2015).

5. Kim, D., Paggi, J.M., Park, C., Bennett, C. & Salzberg, S.L. Graph-based genome alignment and genotyping with HISAT2 and HISAT-genotype. *Nature biotechnology* **37**, 907-915 (2019).

6. Liao, Y., Smyth, G.K. & Shi, W. featureCounts: an efficient general purpose program for assigning sequence reads to genomic features. *Bioinformatics* **30**, 923-30 (2014).

7. Love, M.I., Huber, W. & Anders, S. Moderated estimation of fold change and dispersion for RNA-seq data with DESeq2. *Genome biology* **15**, 1-21 (2014).

8. Pertea, M. *et al.* StringTie enables improved reconstruction of a transcriptome from RNA-seq reads. *Nat Biotechnol* **33**, 290-5 (2015).

9. Langmead, B. & Salzberg, S.L. Fast gapped-read alignment with Bowtie 2. *Nat Methods* **9**, 357-9 (2012).

10. Open2C *et al.* Cooltools: Enabling high-resolution Hi-C analysis in Python. *PLoS Comput Biol* **20**, e1012067 (2024).

11. Lopez-Delisle, L. *et al.* pyGenomeTracks: reproducible plots for multivariate genomic datasets. *Bioinformatics* **37**, 422-423 (2021).

12. Lan, X. & Pritchard, J.K. Coregulation of tandem duplicate genes slows evolution of subfunctionalization in mammals. *Science* **352**, 1009-13 (2016).

13. Hao, F. *et al.* Chromosome-level genomes of three key Allium crops and their trait evolution. *Nat Genet* **55**, 1976-1986 (2023).

14. Xiao, P.X. *et al.* High-quality assembly and methylome of a Tibetan wild tree peony genome (Paeonia ludlowii) reveal the evolution of giant genome architecture. *Hortic Res* **10**, uhad241 (2023).

15. Langfelder, P. & Horvath, S. WGCNA: an R package for weighted correlation network analysis. *BMC Bioinformatics* **9**, 559 (2008).

16. Sun, L. *et al.* Mapping nucleosome-resolution chromatin organization and enhancer-promoter loops in plants using Micro-C-XL. *Nat Commun* **15**, 35 (2024).

17. Zhao, L. *et al.* Integrative analysis of reference epigenomes in 20 rice varieties. *Nat Commun* **11**, 2658 (2020).
